# Effective control of early Zika virus replication by Dengue immunity is associated to the length of time between the 2 infections but not mediated by antibodies

**DOI:** 10.1371/journal.pntd.0008285

**Published:** 2020-05-28

**Authors:** Crisanta Serrano-Collazo, Erick X. Pérez-Guzmán, Petraleigh Pantoja, Mariah A. Hassert, Idia V. Rodríguez, Luis Giavedoni, Vida Hodara, Laura Parodi, Lorna Cruz, Teresa Arana, Melween I. Martínez, Laura White, James D. Brien, Aravinda de Silva, Amelia K. Pinto, Carlos A. Sariol

**Affiliations:** 1 Department of Microbiology and Medical Zoology, University of Puerto Rico-Medical Sciences Campus, San Juan, Puerto Rico, United States of America; 2 Unit of Comparative Medicine, Caribbean Primate Research Center, University of Puerto Rico-Medical Sciences Campus, San Juan, Puerto Rico, United States of America; 3 Department of Molecular Microbiology & Immunology, Saint Louis University School of Medicine, Saint Louis, Missouri, United States of America; 4 Host Pathogen Interactions Program, Texas Biomedical Research Institute, San Antonio, Texas, United States of America; 5 University of North Carolina Chapel Hill, North Carolina, United States of America; 6 Department of Internal Medicine, University of Puerto Rico-Medical Sciences Campus, San Juan, Puerto Rico, United States of America; Faculty of Science, Ain Shams University (ASU), EGYPT

## Abstract

Little is known about the contribution of virus-specific and cross-reacting antibodies (Abs) or the cellular immune response generated by a primary dengue (DENV) infection on the course of a secondary zika (ZIKV) infection *in vivo*. Here we show that the length of time between DENV/ZIKV infections has a qualitative impact on controlling early ZIKV replication. Depletion of DENV2-specific Abs in sera confirmed that those type-specific Abs do not contribute to ZIKV control. We show that the magnitude and durability of the neutralizing antibodies (nAbs) induced by a secondary ZIKV infection is modest compared to the response induced after a secondary heterologous DENV infection. Our *in vivo* results are showing a complex interplay between the cellular and innate immune responses characterized by a high frequency of plasmacytoid dendritic cells (pDC) correlating with an increase in the frequency of DENV antigen specific T cells and a significant control of ZIKV replication which is time dependent. Taken together, our results suggest that early after ZIKV infection other mechanisms such as the innate and cellular immune responses may play a predominant role in controlling ZIKV replication. Regardless of the time elapsed between infections there was no evidence of *in vivo* antibody-dependent enhancement (ADE) of ZIKV by DENV immunity. These findings have pivotal implications while interpreting ZIKV pathogenesis in flavivirus-experimented populations, diagnostic results interpretation and vaccine designs and schedules among others.

## Author summary

From our previous work in non-human primates and others using humans, we believe that previous DENV immunity confers some degree of protection against ZIKV infection. However, at least two highly relevant questions remain unanswered. One is precisely if the time between primary DENV and a subsequent ZIKV infections may play a role in the degree of protection conferred by DENV immunity. The second question is related to the mechanisms of cross-protection. In this work we provide evidences that a period of 12 months between DENV and ZIKV infections has a significant impact controlling ZIKV replication compared to a shorter period of 3 months. We also provide evidences that the pre-existing DENV Abs play no role controlling early ZIKV replication. Our results strongly suggest that the mechanisms controlling ZIKV replication are related to the complex interaction between the innate and the cellular immune responses. Our results have significant implications for vaccine design and schedules.

## Introduction

Zika virus (ZIKV) spread in the Americas has been linked to unique severe adverse outcomes such as fetal loss [1], congenital Zika syndrome (CZS) [2], Guillain-Barré syndrome (GBS) [3], and rare cases of encephalopathy [4], meningoencephalitis [5], myelitis [6], uveitis [7], and severe thrombocytopenia [8]. ZIKV is mainly transmitted through the bite of *Aedes aegypti*, the same vector implicated in dengue (DENV) infection but there are however other routes of infection including sexual contact [9–11] and vertical transmission [12–14] that increase the spread of the virus. ZIKV and DENV circulate on same geographic areas [15, 16] posing a challenge in different areas like diagnosis, epidemiology, vaccine design and pathogenesis.

While ZIKV exists as a single serotype, DENV has four related but antigenically different serotypes. Prior exposure to a single DENV serotype predisposes individuals to severe disease upon a secondary heterologous DENV infection, and the time interval between infections has been considered as a risk factor [17]. A short interval of time between homologous or heterologous DENV infections usually results in protection from disease, while an extended period of time is associated with the potential for severe dengue [18–20], due to either cross-reactive non-neutralizing antibodies (Abs) [21, 22] and cross-reacting T cells [23–25]. However, the association of previous flavivirus exposure at any time before ZIKV infection with the severe adverse outcomes of the infection is still unclear.

Previously, we showed that a primary DENV infection (>2 years) does not result in an increase in ZIKV viremia or pathogenesis in a non-human primate model (NHP) [26]. Interestingly, DENV-immune animals showed a shorter viremic period compared to DENV-naive macaques. Our previous results further suggested that the cellular immune response in DENV-experimented individuals may play a key role limiting ZIKV infection [26]. Our results on the potential protective role of pre-existing DENV-immunity against ZIKV infection were validated by subsequent NHP works [27, 28] and more recently with results from human cohorts [29, 30]. However, little is known about the molecular and immune mechanisms behind DENV and ZIKV interactions. It has been shown that primary DENV infections do not induce durable ZIKV cross-neutralizing Abs [31, 32] but the contribution of previous DENV cross-reacting Abs to controlling early ZIKV replication *in vivo* and their impact in ZIKV pathogenesis remains unclear. One of the advantages of NHP models in contrast to human cohorts is that they allow the control of external factors, such as the exact moment of infection and the amount of the administered viral inoculum [33]. Furthermore, the similarity of their competent immune system with humans is critical for understanding the mechanisms behind disease pathogenesis.

By using rhesus macaques with primary DENV infections at two different points of time, followed by a secondary ZIKV infection, we found that the length of time separating the infections has a qualitative impact on controlling ZIKV replication. It is known that ZIKV-specific Abs appear in circulation a few days after symptom onset [34] and are responsible for most of the neutralizing ZIKV activity [32]. However, the extent of the contribution of DENV-Abs early after ZIKV infection *in vivo* remains uncertain. For this reason, we looked at the contribution of the humoral immune response during early ZIKV replication. Although cross-binding Abs were detected, previous exposure to DENV has no impact on ZIKV neutralization. That fact was confirmed by depleting DENV2-specific Abs in sera. Lastly, no evidence of ZIKV enhancement associated with prior DENV exposure was observed in this study.

In addition, we demonstrate that a period of time of at least 12 months between DENV and ZIKV infections results in a significant increase in the frequency of the dendritic plasmacytoid cells (pDCs) in the first days after ZIKV infection and with a trend to higher frequency of interferon gamma (IFNγ) or CD107a+ CD4+ T cells. Taken together, these data suggest that early after ZIKV infection the cellular immune response, and not Abs, may play a predominant role in controlling ZIKV replication. This study furthers our understanding of ZIKV pathogenesis in flavivirus-experimented populations and is a major contribution to the field of virus-virus interactions and future vaccine designs.

## Results

### Rhesus macaque cohorts and sample collection

**Fig 1:**
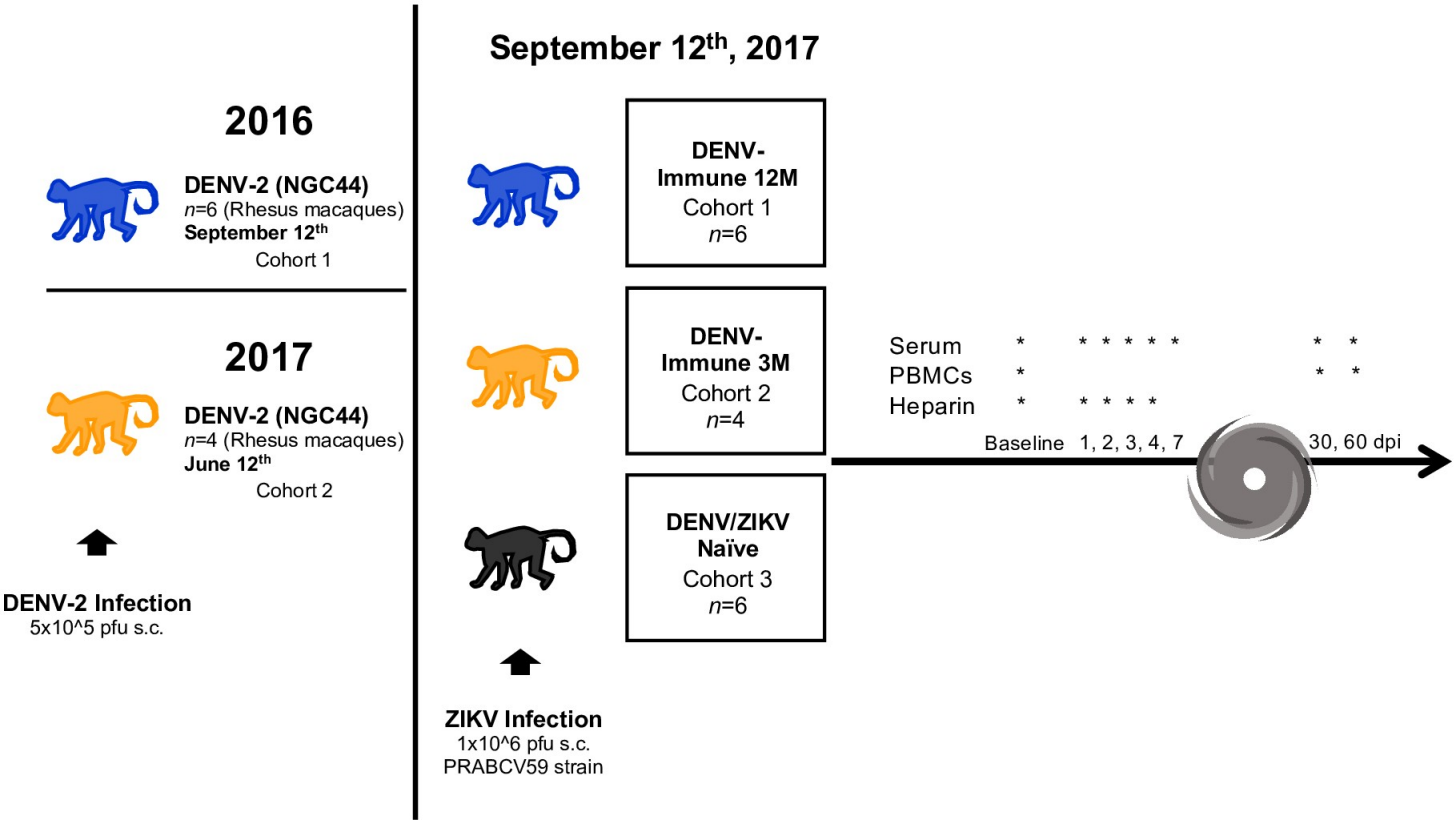
Experimental design of ZIKV infection in DENV-immune and naïve macaques. Two cohorts of rhesus macaques (*Macaca mulatta*) were exposed to DENV-2 (5 x 10^5^ pfu s.c.) at different timepoints. Both cohorts were exposed to ZIKV strain PRABCV59 (1 x 10^6^ pfu s.c.) on September 12^th^, 2017, along with a third cohort composed of ZIKV and DENV naïve animals (n = 6). ZIKV infection was performed 12 months after DENV infection for cohort 1 (n = 6), and 3 months after DENV infection for cohort 2 (n = 4). Serum was collected at baseline and days 1 through 7 post ZIKV infection (p.i.). Sample collection was interrupted by Hurricane María’s impact, and resumed on day 30 p.i. PBMCs could only be obtained on baseline, day 30 and 60 p.i., while heparinized whole blood was collected on baseline and days 1 through 3 p.i. Additionally, urine was collected on baseline and days 2, 4 and 6 p.i.

Six rhesus macaques (*Macaca mulatta*) were infected with 5 x 10^5^ pfu s.c. of DENV-2 New Guinea 44 in 2016 (cohort 1 in Fig 1). In 2017, four rhesus macaques were infected with the same virus strain and pfu (cohort 2 in Fig 1). In addition, a control group, composed by six flavivirus-naïve rhesus macaques was added (cohort 3 in Fig 1). All three cohorts were infected with 1 x 10^6^ pfu s.c. ZIKV PRVABC59 on the same day, defining exposure time between infections for cohorts 1 and 2 as 12 months or middle convalescent and 3 months or early convalescent, respectively (Fig 1). Prior to the challenge with ZIKV, all sixteen animals were put through a quarantine period of forty days. Fig 1 also denotes the unexpected setback that Hurricane María brought to our work plan. Sample collection programmed from days 7 to 29 p.i. was interrupted due to inability of access and/or lack of power at the CPRC facilities, University of Puerto Rico, San Juan, Puerto Rico.

### Clinical status and laboratory results

To determine how a previous DENV infection affects the clinical status of non-human primates after infection with ZIKV, day 0 (baseline), day 6 p.i. and day 30 p.i. were compared in terms of complete blood count. All sixteen animals belonging to this study were continuously monitored and evaluated twice daily for evidence of disease or injury. All animals were inside the range in terms of weight and similar of age (S1A and S1B Fig). No variations were detected in daily rectal temperature (S1C Fig). All cohorts had a drop in white blood cell counts (WBC) by day 6 p.i. that returned to near baseline levels by day 30 p.i. (S2A Fig). No significant variations were noticed in platelet counts (PLT) for any of the cohorts (S2B Fig). Although no differences were detected between groups, monocytes (MON) were significantly higher in the naïve animals on day 6 p.i. compared to their baseline levels (P<0.05; mean diff.: 0.25, CI95%: 0.00 to 0.46) (S2C Fig). This was not observed in the DENV pre-exposed animals.

### ZIKV RNAemia is affected by the longevity of previous DENV immunity

**Fig 2:**
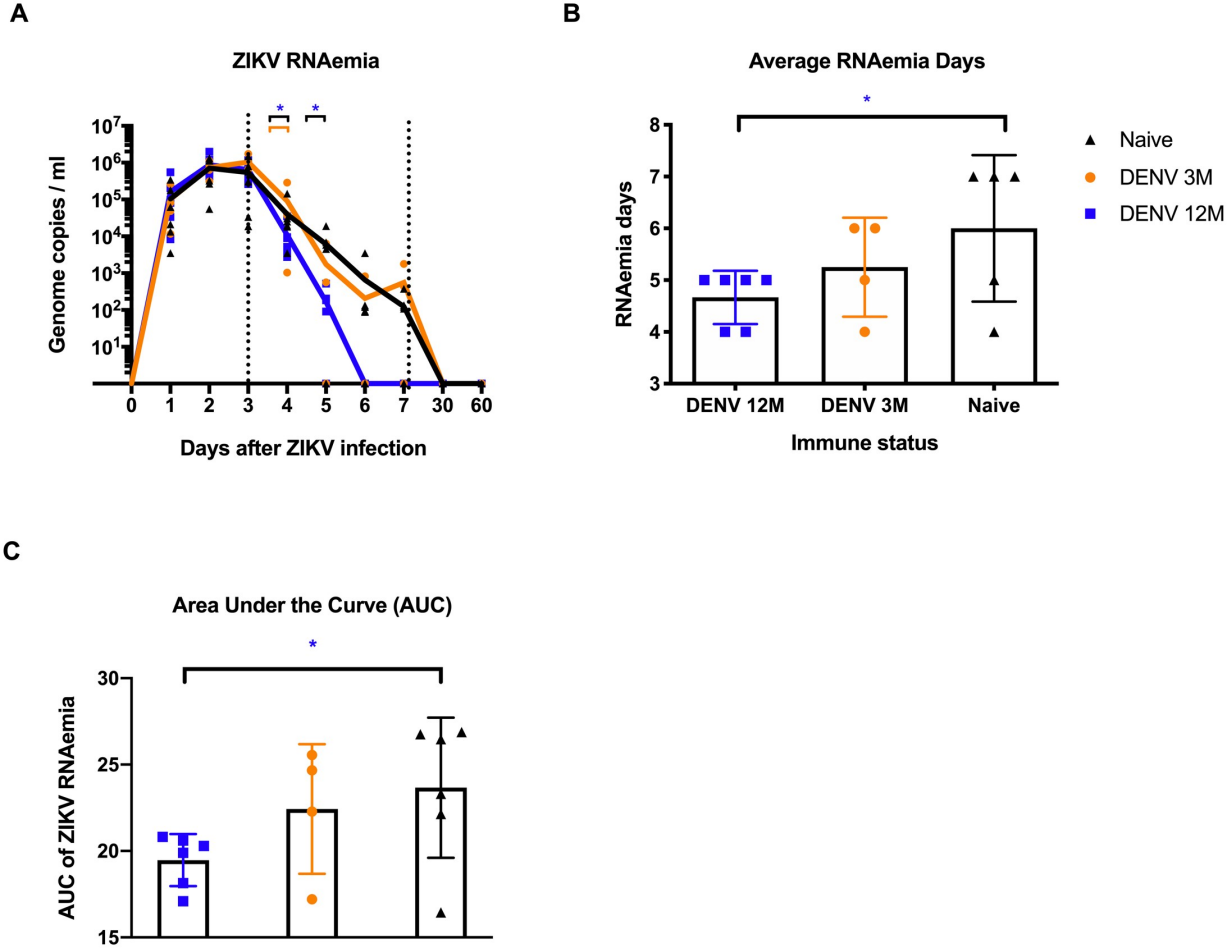
Zika RNA kinetics in serum and RNAemia days per cohort. RNAemia is affected by the period of time between exposures. In all panels, animals exposed to DENV 12 months before ZIKV infection are in blue, while animals exposed to DENV 3 months before are in orange. Naïve animals are in black. (A) ZIKV genome copies/mL in serum. ZIKV replication was detected in serum during the first 7 days after infection, and then on days 30 and 60 p.i. Statistically significant differences were observed using unpaired multiple t tests (*P<0.05). Genome copies per mL are shown logarithmically. (B) Average RNAemia days were calculated using the following formula: total viremia days divided by total possible viremia days and are expressed as percentage. The obtained values were placed in a contingency table. (C) Area under the curve (AUC) was calculated for individual values. Statistically significant differences of viremia days were calculated using a two-sided Fisher’s exact test and multiple t tests (*P<0.05). Colored stars represent a significantly different group, while colored lines represent the group that it is compared to.

To determine if previous immunity to DENV enhances or reduces ZIKV replication, and how it changes depending on the convalescence period, ZIKV RNAemia was measured in serum and urine using qRT-PCR. During the first 3 days p.i., viral RNA detection increased similarly in all groups (Fig 2A). Of note, DENV-middle convalescent animals had significantly lower peak viremia on day 4 p.i. compared with the rest of the animals (P = 0.042 vs. naïve and P = 0.019 vs. DENV 3M group). By day 5 p.i. all three groups had two animals with undetectable viremia. However, the set-point viremia in the four animals from DENV 12M group was significantly lower compared to the four animals having viremia in the naïve group (P = 0.039) (Fig 2A and Table 1). By days 6 and 7 p.i., there was no viral RNA detection in the DENV-middle convalescent group, while DENV 3M animals showed a trend towards an intermittent viremia and most of the naïve animals still had detectable viral RNA. By days 30 and 60 p.i. all animals tested negative for ZIKV (Fig 2A and Table 1).

**Table 1:** ZIKV RNAemia days and neutralizing antibody titers of naïve and DENV-immune macaques. ZIKV RNA detection was consistent in all groups during the first 4 days post infection (p.i.). Peak viremia occurred on day 3 p.i. Cohort 1 animals had no detection of ZIKV RNA in serum by day 6 p.i. Mean viremia days per group was calculated using days with detectable RNAemia divided by the number of animals in each group.

ID	Immune History	RNAemia (Log10 genome copies/mL) (ZIKV PRNT60) Post-ZIKV Infection*									Days	
		1	2	3	4	5	6	7	30	60	Total	Mean
BS97	1° DENV-2 12 months	5.742 (**<20**)	6.298	6.071 (**<20**)	3.626	1.956 (**40**)	0.0 (**40**)	0.0 (**80**)	0.0 (**5120**)	0.0 (**2560**)	28	4.67
0P1		4.904 (**<20**)	5.725	5.448 (**<20**)	3.947	2.715 (**20**)	0.0 (**20**)	0.0 (**80**)	0.0 (**640**)	0.0 (**640**)		
5O8		4.522 (**<20**)	5.631	5.584 (**<20**)	3.978	0.0 (**20**)	0.0 (**160**)	0.0 (**320**)	0.0 (**640**)	0.0 (**320**)		
1O1		5.303 (**<20**)	6.079	5.584 (**<20**)	4.542	2.264 (**40**)	0.0 (**20**)	0.0 (**80**)	0.0 (**640**)	0.0 (**640**)		
4P0		5.303 (**<20**)	5.631	5.419 (**<20**)	3.716	2.311 (**<20**)	0.0 (**20**)	0.0 (**40**)	0.0 (**2560**)	0.0 (**2560**)		
7O5		3.922 (**<20**)	5.766	5.930 (**<20**)	3.436	0.0 (**20**)	0.0 (**80**)	0.0 (**80**)	0.0 (**2560**)	0.0 (**640**)		
MA123	1° DENV-2 3 months	4.913 (**<20**)	5.495	6.240 (**<20**)	3.012	0.0 (**<20**)	0.0 (**20**)	0.0 (**40**)	0.0 (**2560**)	0.0 (**320**)	21	5.25
MA023		5.419 (**<20**)	5.922	5.815 (**<20**)	4.575	0.0 (**20**)	0.0 (**<20**)	3.252 (**80**)	0.0 (**2560**)	0.0 (**1280**)		
MA029		4.684 (**<20**)	6.049	6.041 (**<20**)	4.568	2.748 (**<20**)	2.915 (**20**)	0.0 (**40**)	0.0 (**1280**)	0.0 (**1280**)		
MA062		4.064 (**<20**)	5.806	5.820 (**<20**)	5.460	3.802 (**<20**)	0.0 (**<20**)	2.639 (**80**)	0.0 (**2560**)	0.0 (**640**)		
MA067	NaÏve	5.255 (**<20**)	6.049	5.488 (**<20**)	4.260	4.281 (**<20**)	1.968 (**20**)	2.09 (**40**)	0.0 (**1280**)	0.0 (**1280**)	37	6.16
MA068		3.546 (**20**)	4.742	4.271 (**<20**)	3.542	0.0 (**<20**)	2.120 (**<20**)	0.0 (**40**)	0.0 (**640**)	0.0 (**1280**)		
BZ34		4.795 (**<20**)	6.071	6.176 (**<20**)	4.481	3.766 (**<20**)	1.946 (**20**)	2.037 (**40**)	0.0 (**2560**)	0.0 (**640**)		
MA141		5.536 (**<20**)	6.123	5.907 (**<20**)	4.296	0.0 (**20**)	2.079 (**<20**)	2.127 (**40**)	0.0 (**2560**)	0.0 (**1280**)		
MA143		4.127 (**<20**)	5.510	5.754 (**<20**)	5.158	3.657 (**<20**)	0.0 (**20**)	0.0 (**20**)	0.0 (**2560**)	0.0 (**1280**)		
MA085		4.324 (**<20**)	5.428	4.527 (**<20**)	4.401	3.835 (**<20**)	3.545 (**20**)	2.573 (**40**)	0.0 (**2560**)	0.0 (**640**)		

*ZIKV Neutralizing antibodies were tested at baseline and days 3, 5, 6, 7, 30 and 60.

We defined average RNAemia days as the days with detectable viremia out of all possible days with detectable viremia, taking into account the number of animals per group, during the collection period. The animals exposed to DENV 12 months earlier had the least viremia days in comparison with the naïve group, and the difference was statistically significant (P<0.05) (Fig 2B). Likewise, we observed a significant difference when comparing animals from cohort 1 and control based on comparison of the area under the curve (AUC) (P = 0.039) (Fig 2C). Lastly, ZIKV RNA in urine was measured using qRT-PCR, but only one animal from the DENV 3M group (MA023) had detectable levels at day 6 p.i. (S3 Fig). These results suggest that a previous infection with DENV contributes to an earlier and significant control of ZIKV viremia in a subsequent infection, but only if at least 12 months (a middle convalescence period) have passed between infections.

### Serological profile is modified by the time between the two infections

To assess the impact of previous exposure to DENV at different times in a humoral response against a subsequent ZIKV infection, all sixteen animals were tested for binding Abs against ZIKV and DENV serotypes following ZIKV infection. All three groups had levels of anti-DENV IgM below the cutoff value during the three collection periods, suggesting ZIKV infection did not induce DENV-specific IgM response (S4A Fig). As expected, all DENV immune animals had detectable IgG levels against DENV at baseline (S4B Fig). Anti-DENV IgG levels were confirmed in both DENV-pre-exposed groups and by day 30 experimented a significant expansion compared to their basal levels (P<0.05; mean diff: -0.27, CI95% -0.5145 to -0.02546 P< 0.005; mean diff: -0.3, CI95% -0.4997 to -0.1003 for the DENV 3M and DENV 12M groups respectively). Those cross-reacting Abs were also significantly higher compared to the naïve animals on day 30 (P<0.0001; mean diff.: 0.96, CI95%: 0.61 to 1.30 for DENV 12M vs naïve at 30 days p.i,; P<0.0001; mean diff.: 0.75, CI95%: 0.37 to 1.14 for DENV 3M vs naïve at 30 days p.i.; P<0.0001; mean diff.: 0.88, CI95%: 0.54 to 1.23 for DENV 12M vs naïve at 60 days p.i.; P<0.001; mean diff.: 0.68, CI95%: 0.29 to 1.07 for DENV 3M vs naïve at 60 days p.i.), slowly decreasing by day 60 p.i. (S4B Fig). After a limited increase of the anti-DENV IgG levels on day 30 p.i. the levels rapidly decrease by day 60 in the naïve group. Moreover, by day 30 p.i., DENV-middle convalescent animals showed a strong trend of having higher levels of anti-DENV IgG compared to the DENV-early convalescent animals, although no statistical significance was reached.

As expected, all ZIKV-infected animals developed anti-ZIKV IgM 30 days after the infection (S4C Fig). However, those Abs were early detected only in three animals from the DENV 12M group with two subjects showing a peak on that day (5O8, 1O1). All DENV-immune animals had detectable levels of anti-ZIKV IgG at baseline compared to the naïve animals, suggesting a strong cross-reactivity between previously generated anti-DENV IgG to ZIKV (S4D Fig). DENV-early convalescent animals had significantly higher levels of anti-ZIKV IgG than the other DENV-immune animals (DENV 12M) at baseline (P<0.05; mean diff.: -0.59, CI95%: -1.15 to -0.05). By day 7 p.i., DENV-immune animals had higher levels of anti-ZIKV IgG that increase throughout days 30 and 60 p.i. A similar but slower increase was detected in the naïve group. Nonetheless, all three groups showed a boost in anti-ZIKV IgG levels by day 30 p.i. with a significant expansion at day 60 p.i. for DENV-immune groups (P<0.0001; mean diff.: -1.187, CI95%: -1.848 to -0.525 and P<0.0001; mean diff.: -1.176, CI95%: -1.716 to -0.636 for DENV 3M and DENV 12M groups respectively (S4D Fig). The same scenario can be observed with the naïve group, which experiments a significant expansion in their level of anti-ZIKV IgG by day 60 p.i. compared to day 30 p.i. (P<0.0001; mean diff.: -1.231, CI95%: -1.771 to -0.691).

Only one animal from the DENV-early convalescent group (MA062) had very low but detectable anti-ZIKV NS1 IgG levels at baseline (S4E Fig). All ZIKV-infected animals showed a boost in anti-NS1 levels by day 30 p.i., with DENV-middle convalescent group having significantly higher levels compared to early convalescent and naïve animals (P<0.01; mean diff.: 1.05, CI95%: 0.2608 to 1.836 for DENV 12M vs. DENV 3M; P<0.0001; mean diff.: 2.15, CI95%: 1.45 to 2.859 for DENV 12M vs naïve). These levels decreased by day 60 p.i., and the drop is more dramatic in the pre-immune animals (S4E Fig). Abs against ZIKV EDIII were also measured in order to determine their contribution to humoral immunity and for their known specific contribution to ZIKV neutralization (S4F Fig). Only one animal from the DENV-middle convalescent group showed detectable levels of anti-ZIKV EDIII before ZIKV infection. Anti-ZIKV EDIII levels for all groups slowly increased throughout 30 and 60 days p.i., and the increase at day 60 p.i. was significant for DENV-immune animals with respect to their basal levels (P<0.05; mean diff.: -0.85, CI95%: -1.68 to -0.03 for DENV 12M animals, and P<0.001; mean diff.: -1.26, CI95%: -2.27 to -0.25 for DENV 3M animals). No significant differences were observed among the groups, suggesting that previous exposure to DENV does not have an impact on the generation of cross-reactive Abs against ZIKV EDIII epitopes and that these Abs may have limited contribution to ZIKV neutralization.

### The time between DENV/ZIKV infections modifies the neutralizing profile

To determine the contribution of binding Abs to the neutralization and the impact of a previous DENV infection in a subsequent ZIKV infection in terms of neutralization potential, all three groups were tested using PRNT and FRNT assays against ZIKV and the four DENV serotypes respectively. Neutralization assays were completed for baseline, days 30 and 60 for DENV and ZIKV. To better understand if the previous DENV immunity plays a role in the neutralization of ZIKV early after the infection, we also ran PRNT assays for days 3, 5, 6 and 7. The endpoint titers are showed in Table 1 along with the viremia set point values in order to facilitate a better interpretation of the relationship between both parameters.

**Fig 3:**
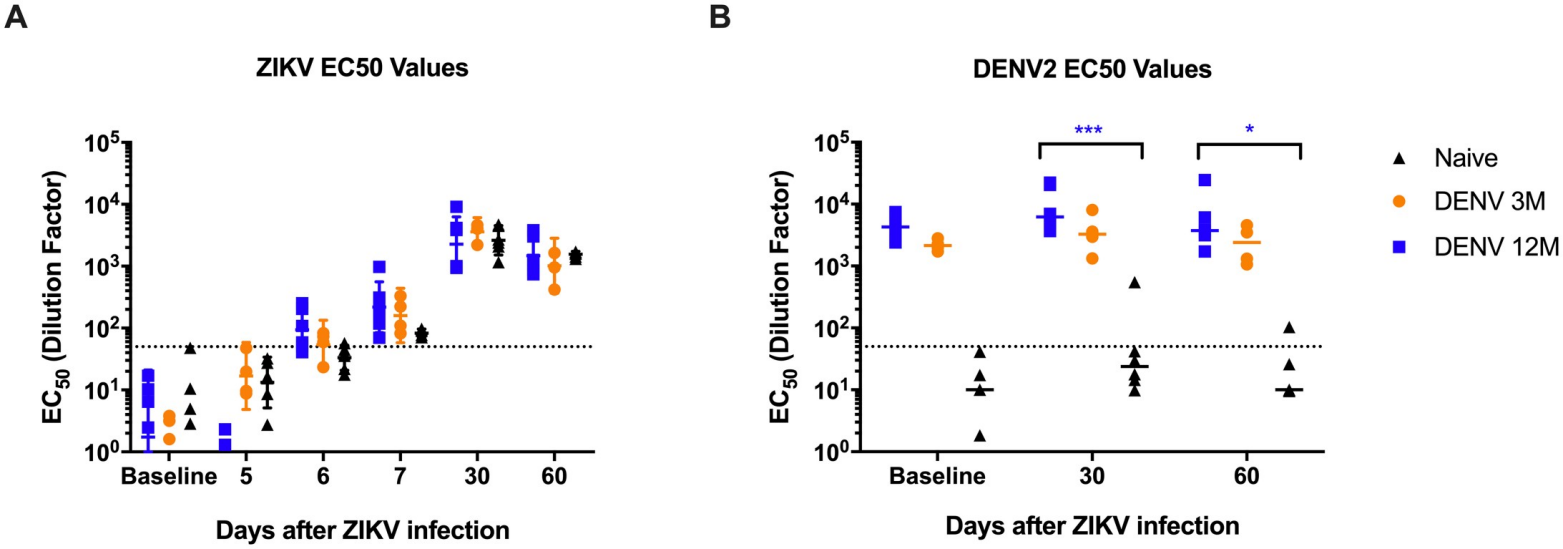
Geometric mean titers of dengue and ZIKV neutralizing antibodies. The 50% effective concentration of neutralizing antibodies was determined. Animals from cohort 1 are shown in blue, animals from cohort 2 are shown in orange and naïve animals from cohort 3 are shown in black in all panels. Dotted line indicates the limit of detection for the assay. Non-neutralizing sera were assigned a value of one-half of the limit of detection for visualization and calculation of the geometric means and confidence intervals. (A) EC50 values of neutralizing antibodies against ZIKV after ZIKV infection. (B) EC50 values of neutralizing antibodies against DENV2 after ZIKV infection. Statistically significant differences among groups were calculated by two-way ANOVA using Tukey’s multiple comparisons test (*P<0.05 and ***P≤0.001). Statistically significant differences among groups were calculated by two-way ANOVA using Tukey’s multiple comparisons test (*P<0.05, **P<0.001, ***P≤0.001 and ****P<0.0001). Colored stars represent a significantly different group, while colored lines represent the group that it is compared to.

The 50% effective concentration (EC) of neutralizing antibodies (nAbs) is shown (Fig 3). As expected, all three groups had low or absent nAbs against ZIKV at baseline (Fig 3A). As early as 6 days after the infection an increase in the neutralizing activity against ZIKV is detected in all groups with a slight non-significant trend to be higher in the DENV 12M group. This increase continues on day 7 with the trend to be higher in both preimmunized groups. By day 30 p.i., neutralizing titers had boosted in the three groups, with DENV-middle convalescent animals showing a slight trend towards higher levels of dilution effective for half-maximum neutralization compared to DENV 3M and naïve groups. These levels decline slightly by day 60 p.i but still maintained a similar relation among groups (Fig 3A).

In order to expand our analysis of the contribution of the humoral immune response to the early viral replication we further looked at the dilution:neutralization capacity relation in samples from days 6 and 7 p.i. As shown in S5 Fig, we identified significant differences, at the highest serum concentrations in the magnitude of the neutralization against ZIKV (only dilutions showing more than 60% of neutralization were considered) on day 6 (P<0.001 and P<0.029 for DENV 12M and DENV 3M vs. naïve respectively at 1:20 dilution and P = 0.0005 and P<0.0001 for DENV 12M vs. DENV 3M and naïve respectively at 1:40 dilution) and day 7 (P<0.0039 and P<0.0001 for DENV 12M vs. naïve at 1:20 and 1:40 dilutions respectively) p.i. However, the role of those early nAbs limiting viral replication is debatable when the relationship between RNAemia and end point neutralizing titers are analyzed together (Table 1).

On the other hand, both DENV immune groups had high levels of nAbs against DENV2 at baseline, which boosted significantly for DENV 12M animals at day 30 p.i. compared to the naïve group (Fig 3B). By day 60 p.i., these nAbs did not decline in neither of the DENV immune groups, and a significant difference was still present for DENV-middle convalescent animals, which suggests that ZIKV infection induced a boost in cross-neutralizing Abs to DENV and the magnitude of the boost depend on the time elapse between DENV and ZIKV infection (P = 0.0006 for day 30 p.i. and P = 0.02 for day 60 p.i.). Only one naïve animal produced low level of nAbs against DENV2 by day 30 p.i. that declined by day 60 p.i. (Fig 3B).

**Fig 4:**
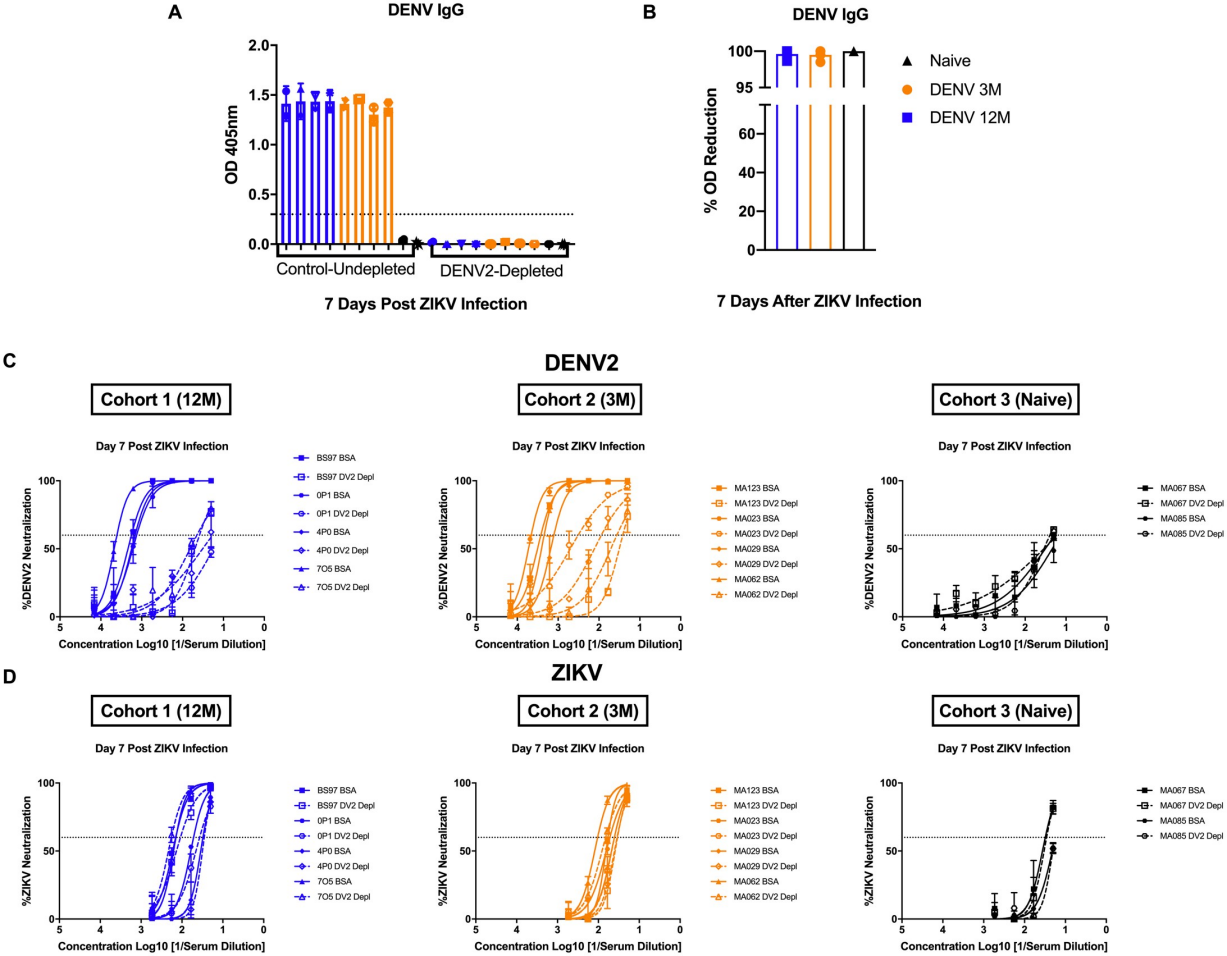
Pre-existing DENV-2 antibodies have no effect on early ZIKV neutralization. Depletion of DENV-2 specific antibodies and its effect on neutralization against DENV-2 and ZIKV is shown. Animals from cohort 1 are shown in blue, animals from cohort 2 are shown in orange and naïve animals from cohort 3 are shown in black in all panels. Dotted line indicates the limit of detection for the assay. Open and closed symbols represent control (undepleted) and depleted samples respectively. (A) Confirmation of DENV-2 antibody depletion by ELISA measuring DENV-IgG binding. (B) Depletion percentage of tested sera shown as optical density (OD) reduction difference between control and depleted samples. (C) DENV neutralization titers of depleted and control sera from day 7 after ZIKV infection are shown. (D) ZIKV neutralization titers of depleted and control sera from day 7 after infection are shown. Due to limited amount of serum, only 4 animals from the DENV-12M group and 2 animals from the naïve group were chosen for serum depletions.

Next, to assess pre-existing DENV Ab responses and their contribution to early ZIKV control, depletion of DENV-2 specific Abs in serum from day 7 p.i. was performed, followed by confirmation by measuring DENV-IgG binding using an ELISA (Fig 4A). Due to limited amount of serum, only 4 animals from the DENV 12M group and 2 animals from the naïve group were chosen for serum depletions. Depletions were more than 98% successful for all tested sera (Fig 4B). As expected, neutralization against DENV-2 was affected by the removal of type-specific Abs (Fig 4C). On the other hand, ZIKV neutralization remained unchanged after depletion of DENV-2 Abs, indicating that ZIKV-specific Abs make up most of the neutralizing force early during ZIKV infection regardless of any pre-existing DENV immunity (Fig 4D).

In order to determine if there were any strain-specific neutralization differences, neutralization assays were performed at 30 days p.i. against two recently circulating contemporary ZIKV strains, ZIKVH/PF/2013 and ZIKVPRVABC-59. No differences in the neutralization magnitude were seen for any group (S6A Fig). When evaluating the neutralizing titers against all four DENV serotypes, we observed a boost in neutralization against all serotypes in all three groups, suggesting that a subsequent ZIKV infection impact the levels of heterologous DENV-neutralizing Abs (S6B Fig). Interestingly 30 days after ZIKV infection there was a non-significant trend to higher neutralizing titers against DENV2 and DENV4 compared to the other two DENV serotypes in the DENV naive group. The hierarchy of neutralizing Abs generated 30 days p.i. was the same for both DENV immune groups (D2>ZIKV>D4>D3>D1), and for the naïve group it was ZIKV>D4>D2>D3>D1 (S6B Fig). Overall, these results show that there are no differences in the magnitude of cross-neutralization of heterologous DENV serotypes between DENV-immune groups in spite of the different times of infection.

### T cell immune response is boosted by the time length between DENV/ZIKV infections

**Fig 5:**
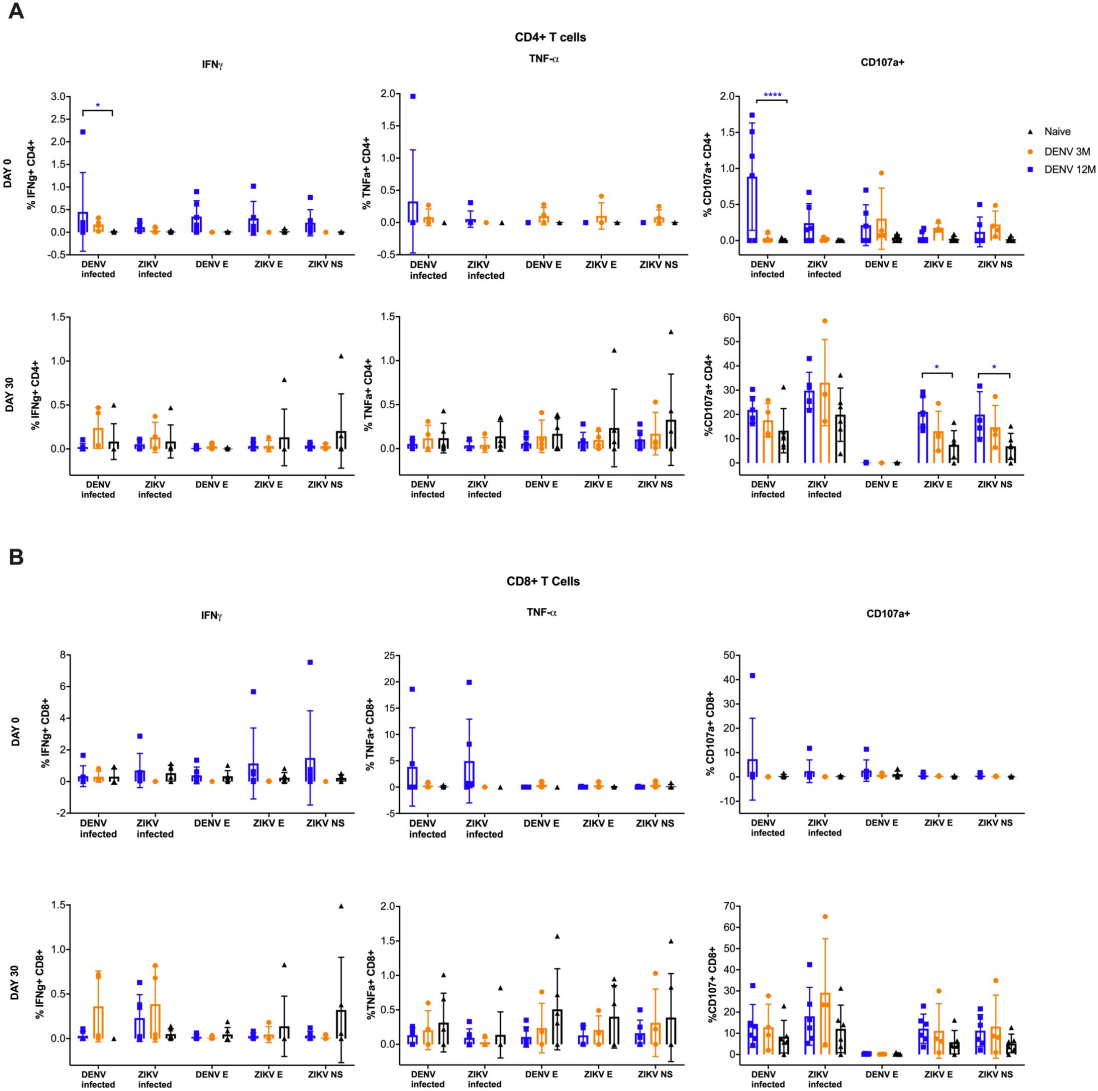
Antigen-specific CD4^+^ and CD8^+^ responses prior and after ZIKV infection. The frequency of the specific response to DENV and ZIKV antigens differs among cohorts. In all panels, animals exposed to DENV 12 months before ZIKV infection are in blue, while animals exposed to DENV 3 months before are in orange. Naïve animals are in black. All percentages shown are subtracted from the unstimulated background. (A) Analysis of CD4 T cell response to different stimuli before (upper panel) and 30 days after ZIKV infection (lower panel). (B) Analysis of CD8 T cell response to different stimuli before (upper panel) and 30 days after ZIKV infection (lower panel). Statistically significant differences among groups were calculated by two-way ANOVA using Dunnett’s multiple comparisons test (*P<0.05 and ****P<0.0001). Colored stars represent a significantly different group, while colored lines represent the group that it is compared to.

To assess if a previous DENV infection has an impact on the T cell response to a ZIKV infection, their effector responses were measured. CD4+ and CD8+ T cells producing IFNγ or TNFα or expressing CD107a in response to various stimuli were assayed (Fig 5). The IFNγ response in the CD4+ T cells from the DENV 12M group before ZIKV infection is remarkable (Fig 5A, upper panel, left). The frequency of these cells in this group was significantly higher in response to the whole inactivated DENV in comparison with the naïve animals (P<0.05) and although not significant, showed a strong trend to have a higher frequency in response to peptides derived from the DENV and ZIKV envelopes and ZIKV non-structural proteins as well compared to the 3M and naïve groups.

DENV 12M animals had also a significantly higher frequency of CD107a+ cells prior to ZIKV infection (P<0.0001; mean diff.: -0.8775, CI95%: -1.25 to -0.503) against the whole DENV antigen (Fig 5A, upper panel, right) while a significant increase in reactivity of CD107a+ CD4+ cells was observed against ZIKV envelope and non-structural antigens on 30 days p.i. (P<0.05; mean diff.: -0.4421, CI95%: -0.8616 to -0.02263 and P<0.05; mean diff.: -0.8775, CI95%: -1.251 to -0.5035, respectively) (Fig 5A, lower panel, right). Nothing remarkable was observed in TNFα expressing CD4+ T cell frequency. In contrast, data from CD8+ T cells denote similar responses between DENV immune animals, with no significant variations compared to the naïve animals (Fig 5B). This suggests that previous DENV immune status preferentially shapes the CD4+ T cells effector responses to a ZIKV infection. Gating strategy is provided as S7 Fig.

### Pro-inflammatory cytokines are not exacerbated by previous DENV immunity

Next, we determined how DENV immunity impacts the cytokine secretion during a subsequent ZIKV infection (S8 Fig). Naïve macaques had significantly higher levels of pro-inflammatory cytokine IFNα on day 1 p.i. compared to the other groups (P<0.05; mean diff.: -145.6, CI95%: -271.7 to -19.37) (S8A Fig). This trend continued through the rest of the collection period, but no other significant differences were detected. DENV 12M animals had seemingly higher levels compared to their DENV 3M counterparts, although no statistical significance was reached. From days 2 to 7 p.i., animals exposed to DENV 12 months before ZIKV infection had a consistently higher expression of IFNγ compared to naïve animals although this difference did not reach statistical significance (S8B Fig). This same group of DENV-middle convalescent animals showed a trend towards higher MIP-1α levels, reaching significant differences in day 5 and 7 p.i. (P<0.01), while naïve animals showed an increase in MIP-1β levels by day 1 p.i. (P<0.01), followed by a sudden drop (S8C and S8D Fig, respectively). Likewise, an increase in IL-1Ra levels, which is considered an inflammatory marker, was detected in naïve animals at day 1 p.i. that is significantly higher than levels in DENV-immune animals (P<0.01; mean diff.: -1723, CI95%: -3060 to -385.1 versus DENV 12M; P<0.01; mean diff.: -1809, CI95%: -3305 to -313.18 versus DENV 3M), but it decreased in the next 24 hours (S8E Fig). A similar event can be observed with CXCL10, where naïve animals had a significant boost by day 1 p.i. in comparison with the two DENV-immune groups, although a dramatic drop occurs on the following 24 hrs (P<0.05; mean diff.: -533, CI95%: -22.69 to -1.98) (S8F Fig). Lastly, DENV 12M animals showed higher levels of circulating perforin, reaching a significant difference compared to the other two groups at day 7 p.i. (P<0.05; mean diff.: -419.9, CI95%: -824.2 to -15.68) (S8G Fig). This result supports a role for this cytolytic protein in early viral clearance.

### Other immune cell subsets frequency is shaped by previous DENV exposure

To establish how previous immunity to DENV shapes the cellular response against a subsequent ZIKV infection, an analysis of the involved cells was performed (S9 Fig). Animals exposed to DENV 3 months earlier had significantly higher frequency of B cells (CD20+) 24 hours before ZIKV infection compared to the other groups (P<0.05; mean diff.: -12.33, CI95%: -22.69 to -1.979 for DENV 3M versus DENV 12M, and P<0.01; mean diff.: 14.77, CI95%: 4.41 to 25.12 for DENV 3M versus naïve group). No other differences between groups were detected, although the trend observed in day 0 is maintained through day 3 (S9A Fig). However, the frequency of activated B cells (CD20+CD69+) was very similar in all three groups (S9B Fig).

**Fig 6:**
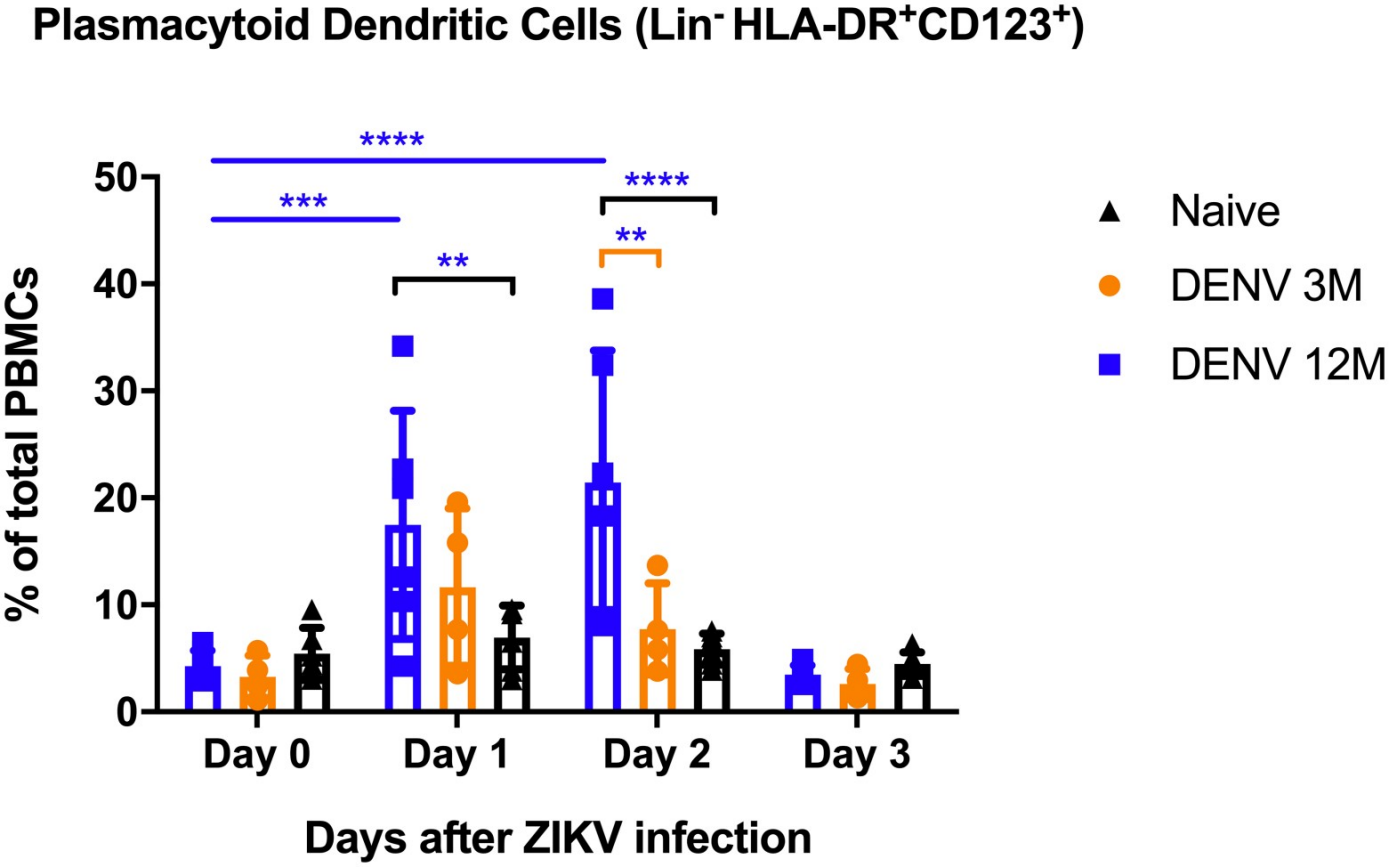
Changes in percentage of plasmacytoid dendritic cells. Percentage of plasmacytoid lineage dendritic cells out of total gated PBMCs. Naïve animals are depicted in black. Animals exposed to DENV 12 months before ZIKV infection are in blue, while animals exposed to DENV 3 months before are in orange. Naïve animals are in black. Differences amongst cohorts in respect with their baseline values were computed by two-way ANOVA using Tukey’s multiple comparisons test (**P<0.01, ***P<0.001 and ****P<0.0001). Colored stars represent a significantly different group, while colored lines represent the group that it is compared to. Same colored lines and stars represent a significant difference compared to their baseline.

Myeloid dendritic cell (mDC) frequency could not be measured on baseline and days 1 and 2 p.i. due to sample quality. Nonetheless, day 3 p.i. data presents no detectable differences in the frequency of dendritic cells of mDC lineage between groups (S10 Fig). In contrast, dendritic cells of plasmacytoid lineage (pDCs) show a significant increase in frequency at days 1 and 2 p.i. in DENV 12M animals in comparison with DENV 3M and naïve animals (P<0.01; mean diff.: 10.54, CI95%: -2.65 to 18.43 for DENV 12M versus naïve animals on day 1 p.i.; for day 2 p.i., P<0.01; mean diff.: 13.68, CI95%: 4.87 to 22.50 for DENV 12M versus DENV 3M and, P<0.0001; mean diff.: 15.57, CI95%: 7.68 to 23.45 for DENV 12M versus naïve animals) (Fig 6). Additionally, this group is the only one where this frequency was significantly elevated on both days compared to their baseline levels (P<0.001 for day 1 p.i. and P<0.0001 for day 2 p.i.). Gating strategy is provided as S11 Fig.

## Discussion

It is well established that exposure to DENV prior to a ZIKV infection results in a qualitative modification of the humoral and cellular immune response to ZIKV in mice, macaques and humans [26, 34–37]. However, we know from sequential DENV infections in humans that the timing between flavivirus exposure can alter the balance between heterologous immune pathogenesis and protection. While there are well structured ongoing studies in human populations living in flavivirus endemic areas [20, 38–41] or following returning travelers from endemic regions, it is difficult to establish the exact time of an infection and more difficult to exactly define the time between two consecutive infections.

Macaques provide a robust model to study the immunological profile after sequential flavivirus infections [26, 28, 42]. Using the macaque model allows among other factors, to normalize the quantity of viral inoculum, the age and sex of the animals exposed, and the timing of the infection. Controlling the timing of exposure between heterologous flavivirus infections allows for the measurement of the impact of time on the immune response to the second infecting agent. These studies are important not only for understanding the impact of the duration of exposure between natural heterologous flavivirus infections in endemic areas but also provide a model to evaluate possible pan-flavivirus protective windows for vaccine candidates.

Previously we have shown that a DENV infection 2.8 years (late convalescence period) prior to ZIKV exposure did not lead to an enhanced ZIKV infection. Moreover, that period of convalescence results in an immune status that trends toward the control of ZIKV viremia and the decrease of liver enzymes after infection. Differences were also observed in B-cell and T-cell activation and cytokine and chemokine profiles [26]. Our previous results have been validated by other groups including a metanalysis using most of the NHP available data [27, 43] and more relevant, in two studies presenting real settings of humans living in DENV-endemic areas [29, 30]. In this work, we aimed to establish the impact of different DENV convalescent periods (3 months or early convalescent period, and 12 months or middle convalescent period) on the immune response after ZIKV infection.

For the DENV 12M group, we identified a significant decline in the viremia set-point on day 4 and 5 p.i., in the average viremia days from days 1 to 7 p.i. and in AUC analysis for viremia, suggesting that the length of time after DENV infection significantly impacts ZIKV replication and most likely its pathogenic effects. Two recent works from human cohorts pointed out the potential role of the length of time elapsed between two infections in the probability to have symptomatic ZIKV infections. Results from a Nicaraguan pediatric cohort showed that when adjusted for age, sex, and recent DENV infection (defined as 1–2 years before ZIKV infection, similar to our DENV-middle convalescence immune group) was significantly associated with a decreased risk of symptomatic ZIKV infection. In addition, the same work showed that prior or recent DENV infection did not affect the rate of total ZIKV infections [29]. Additionally, another work carried out at Pau da Lima community in the city of Salvador, Brazil, showed that the pre-outbreak IgG3 titers against DENV NS1 were positively associated with risk of ZIKV infection but at the same time the presence of IgG1 against same antigen was associated to a decrease in the probability of ZIKV infection [30]. This is highly relevant to our results because IgG3 subclass have been associated to recent DENV infection and are detectable only up to 4 to 6 months after infection, which comprises the period of 3 months of our early-convalescent group showing a trend to control ZIKV replication but not as effective as the DENV-middle convalescent group. However, no specific correlate of protection has been identified for these DENV and ZIKV interactions in those human cohorts. Taking advantage of our model we completed a detailed analysis between the nAbs and the ZIKV RNAemia within the first week of ZIKV infection that otherwise would be difficult to conduct in human cohorts. From days 5 to 7 after ZIKV infection we identified a limited increase in the magnitude of ZIKV neutralization activity in the DENV-middle convalescent group characterized by the presence of low-to-intermediate levels of nAbs. This early expansion of the ZIKV nAbs findings are in agreement with previous works with human monoclonal antibodies (mAbs) confirming an early expansion of the plasmablast response 6 days after secondary DENV [44, 45] and primary ZIKV infections [46]. While no correlation can be made with the viremia on days 5 and 6 p.i., by day 7 a consistent increase in the ZIKV nAbs correlating with the absence of RNAemia was observed only in the animals of DENV 12M group. In order to determine if pre-existing DENV Abs mediate a correlate of protection by controlling early ZIKV replication, we performed depletion of DENV2 specific Abs from day 7 p.i. samples. Our results show that the neutralization against DENV in both DENV-immune groups was abrogated while ZIKV neutralization remains unchanged. This result strongly suggests that pre-existing DENV Abs play no role controlling ZIKV replication during the first week after infection and a contribution in limiting ZIKV pathogenesis and clinical manifestations, as confirmed in NHPs and humans [26–30, 43], is unlikely.

Interestingly, our results on the role of previous DENV Abs on ZIKV replication contrast the findings from our group in a reverse infection scenario, suggesting that low-to-intermediary levels of cross-neutralizing Abs against DENV induced by a previous ZIKV infection may play a role in controlling early DENV RNAemia set point [47].

Additionally, we confirmed the pre-existence of cross-reacting but non-neutralizing Abs to ZIKV in the DENV-immune groups [32, 48]. However, those Abs were significantly higher only in the DENV-early convalescent group compared to the middle convalescent group. This confirms the high frequency of ZIKV cross-reacting Abs during the early DENV convalescence that wane during the middle and late convalescent periods [32, 49]. Cumulatively from our previous results [26] and this current work, we can conclude that the neutralization against ZIKV is very limited or absent in DENV-immune samples, regardless of the convalescence status.

It is known that NS1, along with structural protein E, is one of the main immunodominant targets for B cell response [46, 50]. Here we show that after ZIKV infection, the levels of binding Abs to ZIKV NS1 protein were significantly higher in the group with a middle convalescent period to DENV compared to the other two groups which is in agreement with a trend to higher magnitude of DENV2-neutralizing Abs in that group. Also, in same group we found a significant early expansion in the magnitude of ZIKV neutralization by days 6 and 7 after the infection. These results suggest the presence of more mature memory B cells in that group. Previous studies carried out in human cohorts have suggested that prior DENV exposure could modulate the humoral response to ZIKV [46, 51] while others have inferred that the effect is only modest [31]. It is known that memory B cells are implicated during a secondary infection in a DENV-ZIKV scenario, still it is unknown how or when their maturation takes place in order to modify the response during ZIKV infection. Lastly, earlier works suggested that the magnitude of the humoral response driven by memory B cells may depend on the length of time passed between infections [34]. Here, using an accurate depiction of time elapsed in a NHP model, we show that the length of time between infections does impact the quality of this memory recall.

It is well documented that secondary flavivirus infections, including ZIKV, lead to an increase in cross-reactive Abs and nAbs [32, 34, 48, 49]. Consistent with that, we found a transient but significant expansion in the magnitude of DENV2 cross-reactive Abs in the pre-immune groups 30 days after ZIKV infection with a rapid decline similar to baseline levels by day 60 p.i. DENV cross-neutralizing Abs also show a slight but not significant expansion by day 30 p.i. compared to their baseline values. These results confirm that ZIKV infection contributes to a transient expansion of DENV neutralization which may play a role modifying the course of a subsequent DENV infection in a DENV endemic region. Interestingly, the magnitude of the neutralization against the others 3 DENV serotypes was also boosted but without significant differences among serotypes, regardless of the time between infections. This result is in contrast with our previous work showing that time between ZIKV and DENV infection significantly modify the magnitude of neutralization to the other DENV serotypes suggesting that different immune mechanisms are triggered when the sequence of infections are different [47].

By depleting DENV2 specific Abs from day 7 p.i. samples we confirmed previous results showing that during the convalescence period those Abs do not contribute to ZIKV neutralization [31, 32]. Here we build on existing literature and are adding evidences showing that that scenario is the same regardless of the time elapsed between the two infections (being 3 or 12 months).

In addition our results show that the expansion and durability of the DENV nAbs induced by a secondary ZIKV infection is modest compared to the reported response induced after a secondary heterologous DENV infection [52, 53].

From our work, we can infer a role of the cellular immune response in facilitating the initial significant decrease of ZIKV replication between days 4 to 7 p.i. is very likely. The cytotoxic profile of the CD4+ T cells present 12 months after DENV infection and during heterologous ZIKV challenge correlates with better performance relative to the early (3 months) or late (2.8 years) [26] convalescence periods after primary DENV infection. The role of CD4+T cells in flavivirus infection has been extensively documented [54, 55]. Importantly, Weiskopf et al. and others have shown that DENV CD4+ T cells are readily detectable early following DENV infection, and the frequency of DENV-specific CD107a+ CD4+ T correlate with enhanced protection against DENV disease [56, 57] and play a key role in controlling secondary flavivirus infections [35]. Our work builds on these observations and demonstrates that DENV specific CD4+ T cells isolated one year after DENV infection were highly responsive to the whole DENV virus prior to ZIKV infection (characterized by a significantly higher frequency of IFN-γ production or CD107a expression). Additionally, this group showed a strong trend to higher frequency of CD107a expressing CD4+ T cells in response to the whole ZIKV and of IFN-γ CD4+ T cells after the other stimuli including the whole ZIKV, ZIKV and DENV envelope and ZIKV nonstructural proteins compared to the other two groups. In fact, after 30 days of infection the focus of CD4+ T cells reactivity was ZIKV envelope and non-structural antigens. That switch was a trend, but not significant in animals with an early period of convalescence to DENV compared to the DENV-naïve group. Interestingly, it has been shown that a higher frequency of DENV-specific IFN-γ-producing T cells are associated with subclinical manifestations in children suffering from secondary DENV infection [58]. Notably, the data for CD8+T cells did not recapitulate the observations of the CD4+ T cells. We noted relatively similar responses between CD8+T cells isolated from the 12M DENV immune animals compared to the 3M DENV immune animals. Our finding on CD4+ T cells is consistent with a previous report confirming that pre-existing memory CD4+ T cells (and not CD8+ T cells or Abs) are responsible also for limiting the severity of illness caused by influenza [59]. This suggests that the CD4+ T cell response changed over time, leading to potential differences in disease outcome.

The significant role of T cells in controlling ZIKV replication in animals with a DENV-middle convalescence period before ZIKV infection is reinforced by the significant increase of circulating cytolytic protein perforin at day 7 p.i. We hypothesize that this likely represents T cells acquisition of cytotoxic function [60] in that group compared to the other two groups and correlates with the higher expression of CD107a on the CD4+ T cells isolated from these animals. Previously we confirmed a peak in perforin levels in serum 6 days after ZIKV infection in animals with 2.8 years of previous immunity to DENV [26]. Others have shown that Granzyme B levels in CD4+ and CD8+ T cells peaked between 7 and 10 days post-ZIKV infection [61].

The protective role of the cellular immune response controlling the viral burden of ZIKV in mice has been reported [62, 63]. More recently, mouse models have shown that prior DENV immunity can protect against ZIKV infection during pregnancy, and CD8+ T cells are sufficient for this cross-protection [64]. An isolated but highly relevant finding was that on days 1 and 2 p.i. animals from the DENV 12M group had significant higher levels of pDC frequency in comparison with animals from DENV 3M and naïve groups and with its own baseline levels. It has been shown that pDC levels correlate with DENV severity, with various studies reporting a decrease in pDCs in patients with more serious forms of DENV disease such as Dengue Hemorrhagic Fever (DHF) in comparison with normal DENV or healthy patients [65, 66]. More importantly, De Carvalho and colleagues reported that higher levels of pDCs correlate with lower DENV viremia levels [67]. Other viral infections, like HIV-1 and HCV, lead to an increase in pDCs frequency in long-term survivors, while a decrease has been documented in chronic patients, accompanied by impairment of cytokine-secreting and T cell-activating capacities of pDCs. In addition, during influenza infection pDCs are capable of priming primary and secondary CD4 and CD8 T cell responses *in vitro* and *in vivo* [68]. This is in agreement with the fact that out of the three cohorts in our work, the animals exposed to DENV 12 months before had the most efficient ZIKV viremia clearance.

pDC-mediated mechanisms have been described as key in antiviral responses against DENV [69, 70]. It has been documented that mature pDCs produce large amounts of inflammatory cytokines and chemokines, such as IFN α and IFN β, activating innate and adaptive immune responses during viral infections [69, 71, 72]. Virus-stimulated pDCs are responsible for a wider and faster response, and additionally contribute to expand antigen-specific memory CD4 and CD8 T cells [69]. Thus, their recruitment to lymphoid and some peripheral tissues leads to an ongoing inflammatory response and the activation of lymphocytes by expressing co-stimulatory molecules and in turn, modulate T cell effector functions. Our results are in agreement with this, showing a significant increase of circulating perforin by day 7 p.i. correlating with a higher expression of CD107a and higher frequency of IFN-γ production on CD4+ T cells in animals exposed to DENV 12 months before ZIKV infection.

Altogether, our data reinforces that the activation of the adaptive response against DENV and ZIKV infections involves signals and interactions from pDCs and T cells. This finding on the frequency of pDCs correlating with the reported increase in the frequency of DENV antigen specific T cells and a significant control of ZIKV replication observed only in the DENV 12M group denotes that the complex interplay between the cellular and innate immune responses could be time-dependent.

The uniqueness of our report is that we provide evidence that the magnitude and breadth of flavivirus immunity depends not only on pre-infection immune status but time between exposures, with significantly different protection outcomes. Also, taking advantage of the NHP model, we are providing a dissection of the early events of the protective immune response. We present evidences supporting a limited role, if any, of the pre-existing DENV humoral immune response controlling ZIKV replication. Furthermore, our work indicates an early role of the innate and cellular immunities in such control, allowing a window of transition to the eventual contribution of the humoral immune response.

We acknowledge that to establish the precise role of the T cells immune response controlling ZIKV viremia and pathogenesis, depletion of the specific T cells subsets is advised, and our group is already working on that direction. From our results we cannot anticipate if the effect of previous DENV immunity or the time between DENV and ZIKV infections may have any implications during pregnancy. However, studies addressing the role of DENV Abs in pregnant women infected with ZIKV, one from Italy and one from Brazil, concluded that pre-existence of DENV Abs are not related to an increase of fetal damage [73, 74]. Moreover, the Brazilian study concluded that multitypic DENV infections may protect, rather than enhance, from development of CZS. Despite this, a more recent report from Carvalho et al., published after our work was available as a preprint, concluded that the time elapsed between the more recent DENV epidemics in Brazil and the microcephaly epidemic observed after the introduction of ZIKV was in fact relevant to either provide protection or to increase the risk [75].

More complex studies using a larger number of NHPs and well controlled prospective studies in human populations are needed to elucidate such a relationship [76, 77]. Based on other results from our group, it is possible to argue that the sequence of ZIKV-DENV infections [47] induces a different immunological response—in terms of the neutralization magnitude, cytokine profile and functionality of the cellular immune response—compared to the DENV-ZIKV scenario shown here. However, in both scenarios, the time interval between infections seems to play a critical role in the quality and quantity of the immune response.

Our findings have enormous impact for the epidemiological models anticipating the magnitude of new ZIKV epidemics in DENV endemic areas and are essential for the planning and evaluation of ZIKV and DENV vaccine schedules, design and monitoring.

## Methods

### Viral stock

ZIKV PRVABC59 strain was obtained from ATCC, BEI Resources (Manassas, VA), was used in order to compare results to our previously published data. This ZIKV strain replicates well in rhesus macaques but has a lower viremia peak than ZIKV H/PF/2013 strain. We aimed to use a strain from the recent epidemic in the Americas region. Virus was expanded and titered by plaque assay and qRT-PCR using protocols standardized in our laboratories. DENV-1 Western Pacific 74, DENV-2 New Guinea 44, DENV-3 Sleman 73 and DENV-4 Dominique strains kindly provided by Steve Whitehead (National Institutes of Health, Bethesda, Maryland) were used for neutralization assays. DENV-2 New Guinea 44 strain was also used to infect macaques in September 2016 and June 2017.

### Ethics statement

All procedures were reviewed and approved by the Institute’s Animal Care and Use Committee at Medical Sciences Campus, University of Puerto Rico (IACUC-UPR-MSC) and performed in a facility accredited by the Association for Assessment and Accreditation of Laboratory Animal Care (AAALAC) (Animal Welfare Assurance number A3421; protocol number, 7890116). Procedures involving all study animals were approved by the Medical Sciences Campus, UPR IACUC and were conducted in accordance with USDA Animal Welfare Regulations, the Guide for the Care and use of Laboratory Animals and institutional policies. In addition, steps were taken to lighten sufferings, including use of anesthesia and method of sacrifice if appropriate, in accordance with the recommendations of the Guide for the Care and use of Laboratory Animals (8th edition), Animal Welfare Act and the Public Health Service (PHS) Policy on Humane Care and Use of Laboratory Animals and in accordance with the recommendations of the Weatherall report, “The use of non-human primates in research: http://www.acmedsci.ac.uk/more/news/the-use-of-non-human-primates-in-research/. Macaques were continuously monitored by trained veterinarians at the Animal Research Center and evaluated twice daily for evidence of disease or injury. Feeding and drinking continued normally during this period. All procedures were conducted under anesthesia by intramuscular injection of ketamine at 10–20 mg/kg-1 of body weight, as approved by the IACUC. Anesthesia was delivered in the caudal thigh using a 23-Gauge sterile syringe needle. During the period of the entire study, the macaques were under the environmental-enrichment program of the facility, also approved by the IACUC.

### Immunization and virus challenge of macaques

Young adult rhesus macaques (4–7 years of age) seronegative for DENV and ZIKV were housed in the CPRC facilities, University of Puerto Rico, San Juan, Puerto Rico. For the ZIKV challenge, macaques previously infected with DENV-2 in September 2016 (Cohort 1, n = 6) and June 2017 (Cohort 2, n = 4), and DENV/ZIKV-naïve macaques (Cohort 3, n = 6) were infected subcutaneously in the deltoid area with 500uL of virus diluted in PBS, using a dose of 1 x 10^6^ pfu. All macaques were male. The average age for cohort 1 was 5.1 years (5.6, 4.9, 5.1, 4.9, 5.0 and 5.0 years), 6.8 years for cohort 2 (5.5, 7.75, 7.6 and 6.7 years), and 5.7 years for cohort 3 (6.3, 6.5, 5.8, 4.75, 5.4 and 5.6 years). Weights were taken on Day 0 and every other day during the acute infection period (days 1–6, then at day 30 p.i.). Rectal temperature was taken daily during the acute infection period (days 1–6 and on day 30 p.i.).

### Note on sample collection

The intended schedule was unexpectedly affected by Hurricane María. Sample collection programmed from days 7 to 29 p.i. was interrupted due to inability of access and/or lack of electricity in CPRC facilities in University of Puerto Rico, San Juan, Puerto Rico.

### DENV and ZIKV titration and neutralization assays

For virus titration, Vero81 cells (ATCC CCL-81) at approx. 8.5 x 10^4^ cells/well in 24 well plates with growth medium (Dulbecco’s Modified Eagle’s medium, Thermo Fisher Scientific, with 10% FBS (Gibco), 1% non-essential amino acids (Gibco), 1% HEPES (Gibco), 1% L-glutamine (Gibco) and 1% Pen/Strep (Gibco)). At around 85% confluency (approx. 24 hours later), ten-fold dilutions of virus were prepared in diluent medium (Opti-MEM (Invitrogen) with 2% FBS (Gibco) and 1% antibiotic/antimycotic (HyClone) and added to the wells after removing growth medium. Each virus dilution was added in 100 mL triplicates, and plates were incubated 1 hour at 37C/5%CO2/rocking. After incubation, 1 mL of overlay (Opti-MEM with 1% Carboxymethylcellulose (Sigma), 2% FBS, 1% non-essential amino acids (Gibco), 1% antibiotic/antimycotic (HyClone)) was added to the plates containing viral dilutions, followed by an incubation period at 37C/5%CO2. After 3 to 5 days of incubation (depending of the virus), overlay was washed away with phosphate buffered saline (PBS 1X) and fixed with 80% methanol. For ZIKV, cells were stained with crystal violet after fixing. For DENV, plates were fixed then blocked with 5% non-fat dry milk in PBS and incubated for 1hr/37C/5%CO2/rocking with anti-E mAb 4G2 and anti-prM mAb 2H2 (provided by Dr. Aravinda de Silva), both diluted 1:250 in blocking buffer. Plates were washed twice and incubated 1hr/37C/5%CO2/rocking with horseradish peroxidase (HRP)-conjugated goat anti-mouse antibody (Sigma), diluted 1:1,000 in blocking buffer. Foci were developed with TrueBlue HRP substrate (KPL) and counted. For the Focus/Plaque Reduction Neutralization Test (FRNT/PRNT), sera were diluted two-fold and mixed with approx. 35 foci per plaque-forming units (FFU per p.f.u. per mL) of virus and then incubated for 1hr/37C/5%CO2/rocking. Virus-serum dilutions were added to 24 well-plates containing Vero81 cells as mentioned above, and incubation was continued for 1hr/37C/5%CO2/rocking. After incubation, overlay was added, and the aforementioned procedure was repeated. Mean focus diameter was calculated from approx. 20 foci per clone measured at X5 magnification. Results were reported as the FRNT or PRNT with 60% or greater reduction in DENV or ZIKV foci or plaques (FRNT60 or PRNT60). A positive neutralization titer was designated as 1:20 or greater, while <1:20 was considered a negative neutralization titer.

### qRT-PCR

Viral RNA for real-time PCR assay was extracted from 140 ul of virus isolate (previously tittered as described above) and serum samples using Invitrogen PureLink RNA Mini Kit (Invitrogen, Valencia, CA) as per the manufacturer’s instructions. Real-time RT-PCR (TaqMan) assay-specific primers and probes for ZIKV were designed by Sigma-Aldrich (St Louis, MO) following the protocol developed by the Molecular Diagnostics and Research Laboratory Centers for Disease Control and Prevention (CDC), Dengue Branch at San Juan, PR. RNA from other flaviviruses were included as negative control. For the reaction mixture, 5 ml of RNA was combined with 100 mM primers and 25 mM probe in a 25 ml total volume using Life Technologies SuperScriptIII Platinum assay kit (Life Sciences). Assays were performed in an iCycler iQ Real Time Detection System (Bio Rad, CA). For quantification, a standard curve was generated from ten-fold dilutions of RNA from a known amount of virus.

### ELISA for DENV and ZIKV

Prior to ZIKV challenge, DENV/ZIKV seronegative status of cohort 3 animals was assessed using DENV IgG/IgM and ZIKV NS1 IgG commercial kits (Focus Diagnostics, CA). After ZIKV infection, seroreactivity to DENV was tested using commercial IgG and IgM ELISA kits (Focus Diagnostics, Cypress, CA). ZIKV IgG was assessed with available commercial kits (XpressBio, Frederick, MD and InBios, Seattle, WA respectively). ZIKV-NS1 and ZIKV-EDIII IgG were examined using a commercial kit (Alpha Diagnostic, San Antonio, TX). All tests were performed per the manufacturer’s instructions.

For the measurement of IgM levels against ZIKV, samples were tested using a ZIKV IgM MAC-ELISA assay developed by Aravinda de Silva’s laboratory. Briefly, a 96-well microtiter plate was coated with anti-human IgM (1:50). Following incubation and blocking with 3% non-fat milk, sample dilutions (1:40) were added. Positive and negative controls for ZIKV and DENV were also prepared. Stock C6/36 ZIKV and DENV antigens were diluted (1:2 and 1:3, respectively), and added to the plate. IgM-binding Abs were detected using horseradish peroxidase (HRP)-conjugated mAb (6B6C-1) and TMB substrate. Optical density at 450 nm (OD) values were measured in three separate readings at 5-minute intervals. Results were expressed as mean OD of sample reacted with viral antigen (P)/mean OD of normal human serum reacted with viral antigen (N) and reported as negative (P/N value of <2), presumptive positive (P/N value of >3) or equivocal (2< P/N <3).

Confirmation of DENV-2 Abs depletion was performed by ELISA by coating plate with capture Ab (4G2/2H2 100 ng/well), followed by blocking with 3% nonfat dairy milk and incubating with purified DENV-2 virus. Depleted or control (undepleted) sera was added (1:40 100 ul/well) and IgG-binding Abs were detected using AP-conjugated secondary anti-goat Ab (1:2500). Absorbance was measured at 405 nm.

### Depletion of DENV-2 Abs

A cross-reactive DENV-binding Ab (1M7) was conjugated to M280 Tosylactivated Dynabeads (Invitrogen) at a ratio of 100 μg 1M7 to 5 mg Dynabeads as per the manufacturer’s instructions. Beads were blocked with 1% BSA/PBS for 1 h at 37 °C. Purified DENV-2 (S-16803) was bound to the 1M7-Dynabead mixture at a ratio of 100 μg antigen to 5 mg DENV2-bound Dynabeads for 1 h at 37 °C and fixed using 4% paraformaldehyde. Control beads were incubated with an equal amount of BSA. For depletion, sera were diluted 1:10 and incubated with 5 μg of DENV2-bound Dynabeads in 3 successive rounds at 37 °C for 1 h each, using a magnet to remove Abs bound to beads after each round. Confirmation of DENV-2 Ab depletion was performed by ELISA (see above). After depletions, neutralization titers were determined using a micro neutralization test (MNT) in which serially diluted sera (depleted and undepleted controls) were incubated with ~50 focus forming units of DENV-2 (S-16803) in Dulbecco modified Eagle medium for 1 h at 37 °C. The serum-virus mixtures were added to Vero cells (2 x 10^4^ cells/well) in a 96-well plate and incubated 1 h at 37 °C. After incubation, 1 mL of overlay was added to the plates containing serum-virus dilutions, followed by an incubation period of 48 hours at 37C/5%CO2. After 2 days, cells were fixed with 4% paraformaldehyde, washed with PBS 1X, blocked with 3% nonfat dairy milk and stained with 4G2 and 2H2 mAbs, diluted 1:250 in blocking buffer, followed by incubation with horseradish peroxidase (HRP)-conjugated goat anti-mouse Ab diluted 1:800 in blocking buffer. Foci were developed with TrueBlue HRP substrate (KPL) and counted using an Elispot reader.

### Cellular immune response analysis

Intracellular cytokine staining of PBMCs from rhesus macaques was performed by multicolor flow cytometry using methods similar to those described by Meyer et al. Briefly, PBMC samples were thawed 1 day prior to stimulation. Approx. 1.5 x 10^6^ PBMCs were infected overnight with DENV-2 (NGC44) at a MOI of 0.1 or ZIKV at a MOI of 0.5 in RPMI medium with 5% FBS. The remaining PBMCs were rested overnight as described earlier in 5ml of RPMI with 10% FBS. These PBMCs were then stimulated for 6 h at 37C/5%/CO2 with ZIKV-E peptides (15-mers overlapping by 10 amino acids, 2.5 ug/ml^-1^ per peptide), ZIKV-NS1 protein peptides (15-mers overlapping by 10 amino acids, 475 ng/ml^-1^ per peptide), or DENV-2 E peptides (1.25 ug/ml^-1^), all in the presence of brefeldin A (10 ug/ml^-1^), a-CD107a-FITC (H4A3) (10 ul), and co-stimulated with a-CD28.2 (1 ug/ml^-1^) and a-CD49d (1 ug/ml^-1^). After stimulation, the cells were stained for the following markers: CD4-PerCP Cy5.5 (Leu-3A (SK3), CD8b-Texas Red (2ST8.5H7), CD3-PacBlue (SP34), CD20-BV605 (2H7), CD95-V510 (DX2), CD28.2-PE-Cy5, IFN-γ-APC (B27) and TNF-α-PE-Cy7 (MAB11). The samples were run on an LSRII (BD) and analyzed using FlowJo v10.6.1 (Treestar). Lymphocytes were gated based on their characteristic forward and side scatter pattern, T cells were selected with a second gate on the CD3-positive population, and at the same time CD20 positive cells were excluded. CD8+ T cells were defined as CD3^+^ CD20^-^CD8^+^ and CD4+ T cells as CD3^+^CD20^-^CD4^+^. Cytokine expression was determined by the per cent CD4+ or CD8+ positive cells, and then stained positive for the cytokine IFNγ or TNFα. CD107a+ was also measured in these populations to determine functional cytotoxicity.

### Multiplex cytokine analysis

Sera from rhesus macaques was analyzed for 14 cytokines and chemokines by Luminex using established protocols for Old World primates. Evaluation of interferon alpha (IFNα), interferon gamma (IFNγ), IL-1 receptor antagonist (IL-1Ra), inflammatory protein 1-alpha (MIP-1α, CCL3) and beta MIP-1β (CCL4), perforin and interferon gamma-induced protein 10 (IP-10, CXCL10) were included in this assay.

### Immunophenotyping

Phenotypic characterization of rhesus macaque cellular immune response was performed by multicolor flow cytometry using direct immunofluorescence. Aliquots of 150 ul of heparinized whole blood were directly incubated with a mix of Abs for 30 min. at room temperature. After incubation, red blood cells were fixed and lysed with ACK, and cells were washed three times with PBS. Samples were analyzed using a MACSQuant Analyzer 10 flow cytometer (Miltenyi Biotec, CA). The following Abs were used in this study: CD123-APC (7G3), CD20-FITC (2H7), CD20-PacBlue (2H7), CD69-PE (FN50), KI67-FITC (B56) and CD3-FITC (SP34) from BD-Biosciences; HLA-DR VioGreen (G46.6), CD3-PE-Vio770 (10D12), CD3-APC (10D12) from Miltenyi; CD11c-PE/Cy7 (3.9), CD8-FITC (SK1) and CD8-BV421 (SK1) from Biolegend. For analyses, LYM were gated based on their characteristic forward and side scatter pattern. B cells were defined as CD20^+^CD3^-^CD14^-^. Activation marker CD69 was determined in each different lymphoid cell population. Dendritic cells (DCs) were separated into two populations by the expression of CD123 (pDCs) or CD11c (mDCs) in the HLA^-^DR^+^CD3^-^ CD14^-^CD20^-^ population. Data analysis was performed using FlowJo v10.6.1 (Treestar).

### Statistical methods

Statistical analyses were performed using GraphPad Prism 7.0 software (GraphPad Software, San Diego, CA, USA). For viral burden analysis, the log titers and levels of vRNA were analyzed unpaired multiple t tests and two-way ANOVA. Also, a Chi-squared test was used to analyze a contingency table created from obtained viremia data. The statistical significance between or within groups evaluated at different time points was determined using two-way analysis of variance (ANOVA) (Tukey’s, Sidak’s or Dunnett’s multiple comparisons test) or unpaired t-test to compare the means. The p values are expressed in relational terms with the alpha values. The significance threshold for all analyses was set at 0.05; p values less than 0.01 are expressed as P<0.01, while p values less than 0.001 are expressed as P<0.001. Similarly, values less than 0.005 are expressed as P<0.005. In figures, p values from 0.01 to 0.05 are depicted as *, 0.001 to 0.01 as **, 0.0001 to 0.001 as ***, and lastly, values less than 0.0001 are depicted as ****.

## Supporting information

S1 Fig Vital signs and age of macaques before and after ZIKV infection. Weight distribution per animal cohort. (A) Weight of rhesus macaques is expressed in kilograms (kg). (B) Age of rhesus macaques considered young adults. (C) Rectal temperature (in Celsius) was measured. Animals exposed to DENV 12 months before ZIKV infection are depicted in blue, while animals exposed to DENV 3 months before are in orange. Naïve animals are in black.
(TIF)

S2 Fig Kinetics of hematology and laboratory results. Cell subsets obtained from complete blood count (CBC) tests at baseline, 6 and 30 days p.i. In all panels, animals exposed to DENV 12 months before ZIKV infection are in blue, while animals exposed to DENV 3 months before are in orange. Naïve animals are in black. (A) White blood cells (WBC) total depicted in thou/uL. (B) Platelet (PLT) levels total depicted in thou/uL. (C) Monocyte (MON) kinetics expressed as absolute numbers (x10^6 cells/mL). Statistically significant differences among and within groups were calculated by two-way ANOVA using Tukey’s multiple comparisons test (*P<0.05).
(TIF)

S3 Fig Zika RNA kinetics in urine. ZIKV RNA detection in urine though day 6 p.i. Animals exposed to DENV 12 months before ZIKV infection are in blue, while animals exposed 3 months before are in orange. Naïve animals are colored black. No statistical differences were detected.
(TIFF)

S4 Fig Serological profile of the three cohorts of macaques before and after ZIKV infection. Humoral response was assessed using different commercial and in-house ELISA tests. (A-F) Binding capacity of antibodies from animals with different immune background are shown. Animals from cohort 1 are shown in blue, animals from cohort 2 are shown in orange and naïve animals from cohort 3 are shown in black in all panels. Dotted lines indicate the limit of detection for each test. Statistically significant differences among and within groups were calculated by two-way ANOVA using Tukey’s multiple comparisons test (*P<0.05, **P<0.005 and ****P<0.0001). Colored stars represent a significantly different group, while colored lines represent the group that it is compared to.
(TIF)

S5 Fig Geometric mean titers of dengue and ZIKV neutralizing antibodies. Dilution titers against ZIKV during days 6 and 7 post ZIKV infection. Animals from cohort 1 are shown in blue, animals from cohort 2 are shown in orange and naïve animals from cohort 3 are shown in black in all panels. Dotted line indicates the limit of detection for the assay. Non-neutralizing sera were assigned a value of one-half of the limit of detection for visualization and calculation of the geometric means and confidence intervals. Statistically significant differences among groups were calculated by two-way ANOVA using Tukey’s multiple comparisons test (*P<0.05, **P<0.001, ***P≤0.001 and ****P<0.0001). Colored stars represent a significantly different group, while colored lines represent the group that it is compared to.
(TIF)

S6 Fig Neutralizing response to heterologous DENV serotypes and two different ZIKV strains. PRNT and FRNT assays were performed to determine the effect of previous DENV immunity in a subsequent ZIKV infection, and the neutralizing antibody response against different dengue serotypes and zika strains. In all panels, animals exposed to DENV 12 months before ZIKV infection are in blue, while animals exposed to DENV 3 months before are in orange. Naïve animals are in black. (A) Neutralization against two different ZIKV strains was performed. Dotted lines indicate the limit of detection for the assay. (B) Neutralizing response against heterologous DENV serotypes before and after ZIKV infection.
(TIF)

S7 Fig Gating strategy for T cell response to stimuli. The gating strategy used to select cells expressing TNFa, IFNg and CD107a upon stimulation with various DENV and ZIKV peptides is shown.
(PDF)

S8 Fig Previous exposure to DENV modulates the cytokine and chemokine profiles after ZIKV infection. (A-G) Significant cytokine and chemokine profiles of are depicted in pg per ml^-1^. In all panels, animals exposed to DENV 12 months before ZIKV infection are in blue, while animals exposed to DENV 3 months before are in orange. Naïve animals are in black. Statistically significant differences among groups were calculated by two-way ANOVA using Tukey’s, Sidak’s and Dunnett’s multiple comparisons tests (*P<0.05, **P<0.001 and ***P≤0.0001). Colored stars represent a significantly different group, while colored lines represent the group that it is compared to. Same colored lines and stars represent a significant difference compared to their baseline.
(TIF)

S9 Fig B cells profile before and after ZIKV infection. Frequency of B cells was assessed. In all panels, animals exposed to DENV 12 months before ZIKV infection are in blue, while animals exposed to DENV 3 months before are in orange. Naïve animals are in black. (A) Percentage of total B cells (CD20+) during baseline and days 1 through 3 p.i. (B) Frequency of activated B cells (CD20+ CD69+) during baseline and days 1 through 3 p.i. Comparisons between cohorts were performed by two-way ANOVA using Tukey’s multiple comparisons test (*P<0.05 and **P<0.01). Colored stars represent a significantly different group, while colored lines represent the group that it is compared to.
(TIFF)

S10 Fig Changes in percentage of myeloid dendritic cells. Percentage of myeloid lineage dendritic cells out of total gated PBMCs. Animals exposed to DENV 12 months before ZIKV infection are colored blue, while animals exposed 3 months before are colored orange. Naïve animals are depicted in black. No statistical differences were detected.
(TIFF)

S11 Fig Gating strategy for dendritic cells. Gating strategy used to define dendritic cell subsets.
(TIF)
