# Supplementary figures and images for: Effective control of early Zika virus replication by Dengue immunity is associated to the length of time between the 2 infections but not mediated by antibodies
Source: PLoS Negl Trop Dis. 2020 May 28;14(5):e0008285. doi: 10.1371/journal.pntd.0008285 (PMC7255596; doi:10.1371/journal.pntd.0008285)

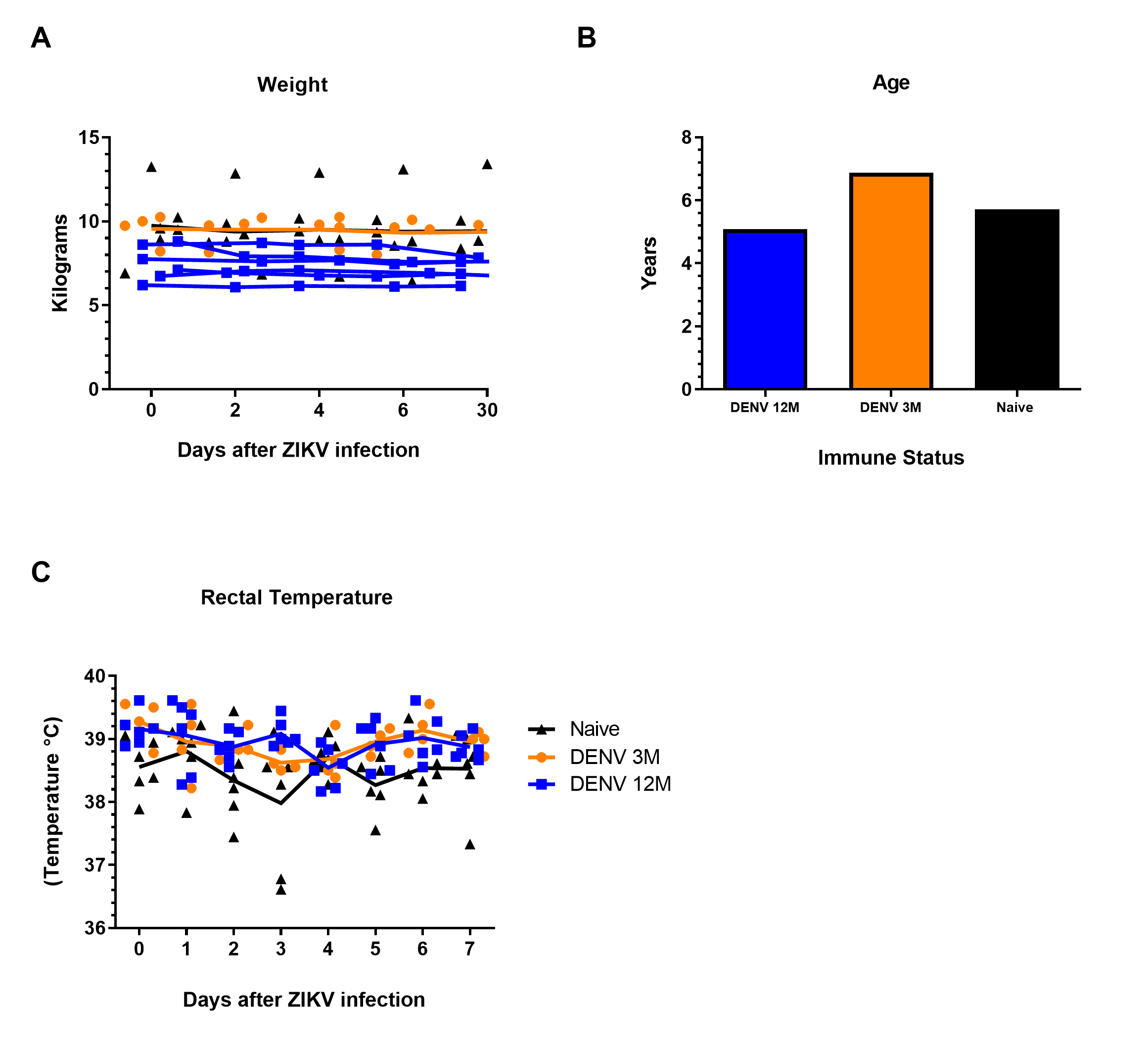

Supplement: S1 Fig — (A) Weight of rhesus macaques is expressed in kilograms (kg). (B) Age of rhesus macaques considered young adults. (C) Rectal temperature (in Celsius) was measured. Animals exposed to DENV 12 months before ZIKV infection are depicted in blue, while animals exposed to DENV 3 months before are in orange. Naïve animals are in black. (TIF) [file pntd.0008285.s001.tif]

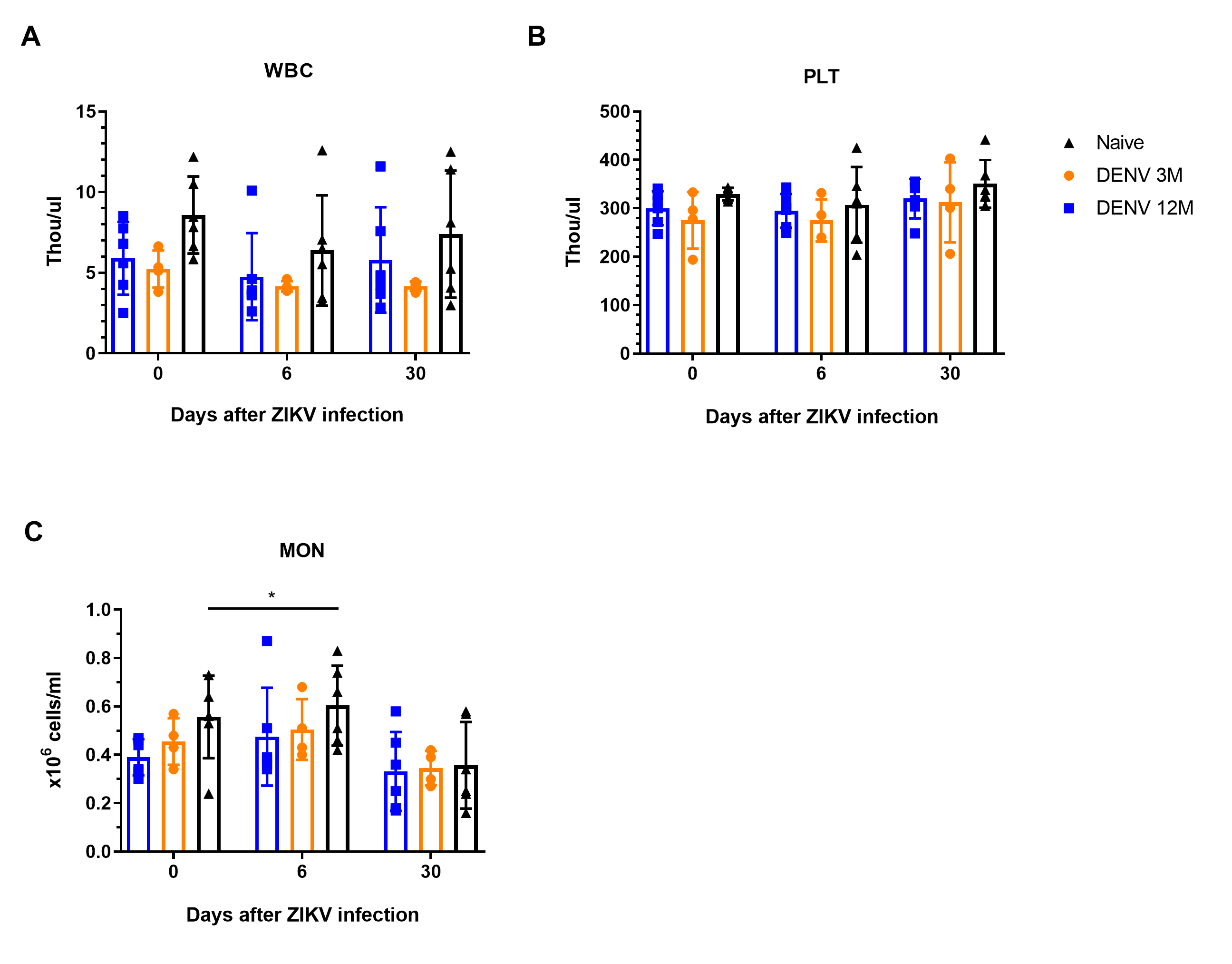

Supplement: S2 Fig — Cell subsets obtained from complete blood count (CBC) tests at baseline, 6 and 30 days p.i. In all panels, animals exposed to DENV 12 months before ZIKV infection are in blue, while animals exposed to DENV 3 months before are in orange. Naïve animals are in black. (A) White blood cells (WBC) total depicted in thou/uL. (B) Platelet (PLT) levels total depicted in thou/uL. (C) Monocyte (MON) kinetics expressed as absolute numbers (x10^6 cells/mL). Statistically significant differences among and within groups were calculated by two-way ANOVA using Tukey’s multiple comparisons test (*P<0.05). (TIF) [file pntd.0008285.s002.tif]

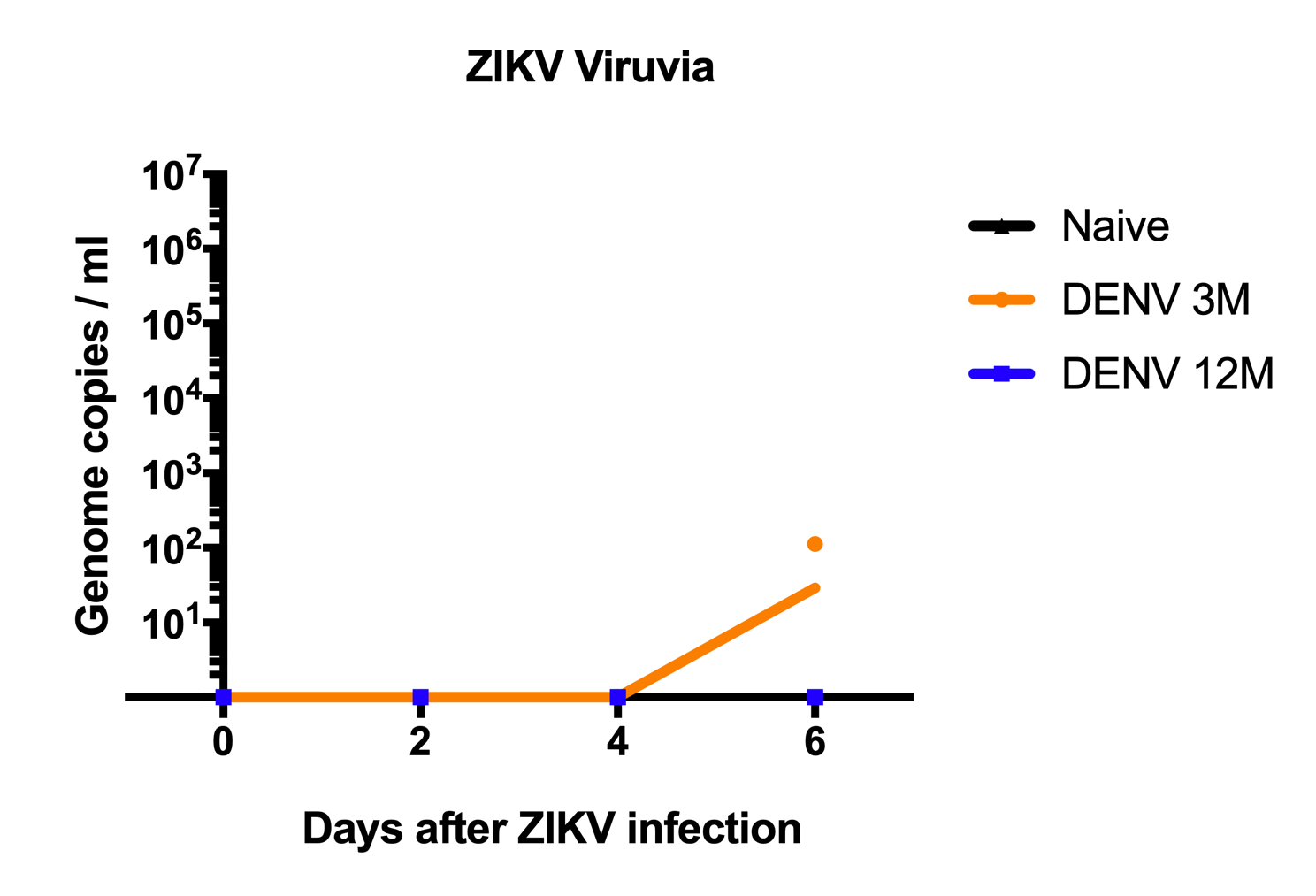

Supplement: S3 Fig — Animals exposed to DENV 12 months before ZIKV infection are in blue, while animals exposed 3 months before are in orange. Naïve animals are colored black. No statistical differences were detected. (TIFF) [file pntd.0008285.s003.tiff]

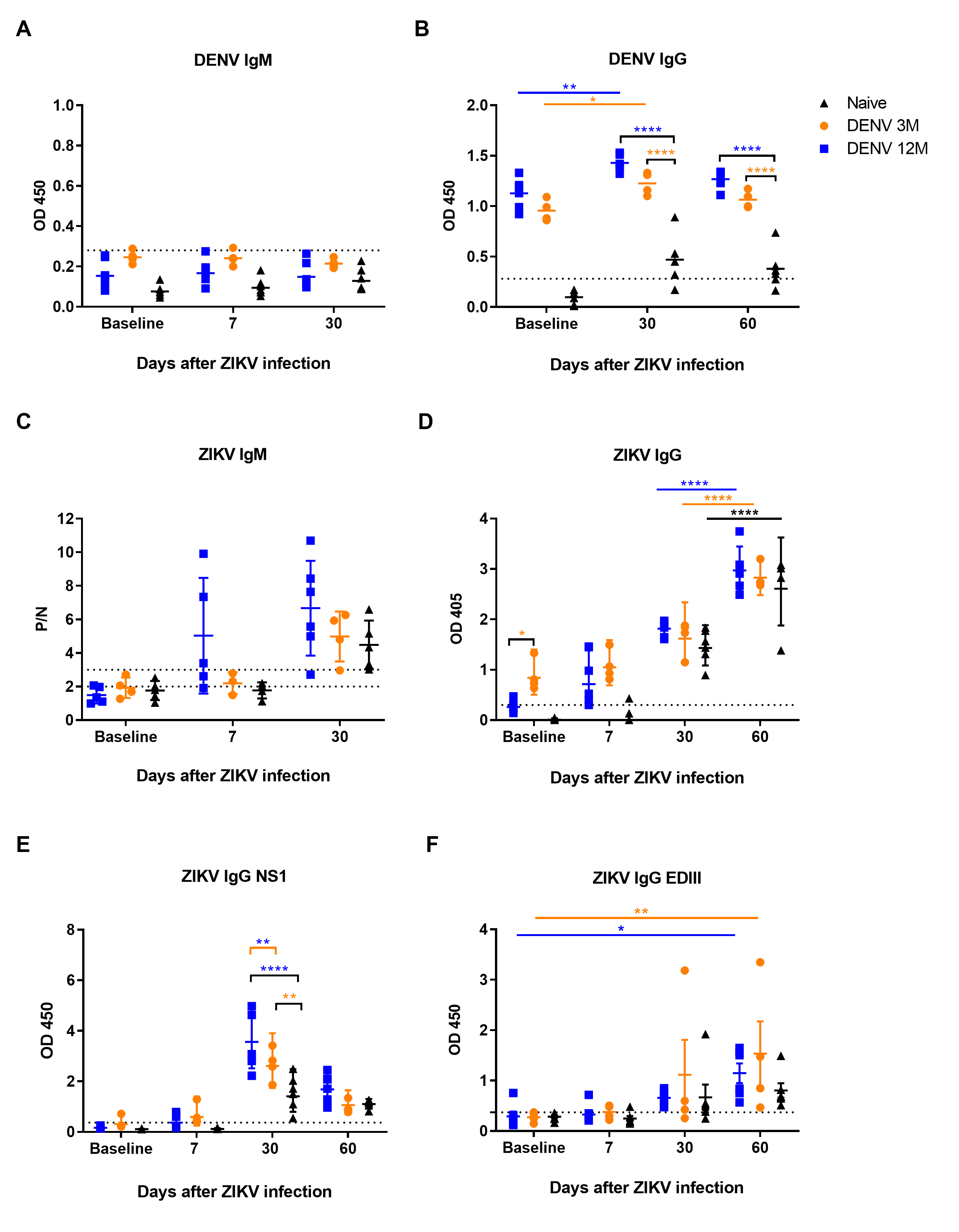

Supplement: S4 Fig — Humoral response was assessed using different commercial and in-house ELISA tests. (A-F) Binding capacity of antibodies from animals with different immune background are shown. Animals from cohort 1 are shown in blue, animals from cohort 2 are shown in orange and naïve animals from cohort 3 are shown in black in all panels. Dotted lines indicate the limit of detection for each test. Statistically significant differences among and within groups were calculated by two-way ANOVA using Tukey’s multiple comparisons test (*P<0.05, **P<0.005 and ****P<0.0001). Colored stars represent a significantly different group, while colored lines represent the group that it is compared to. (TIF) [file pntd.0008285.s004.tif]

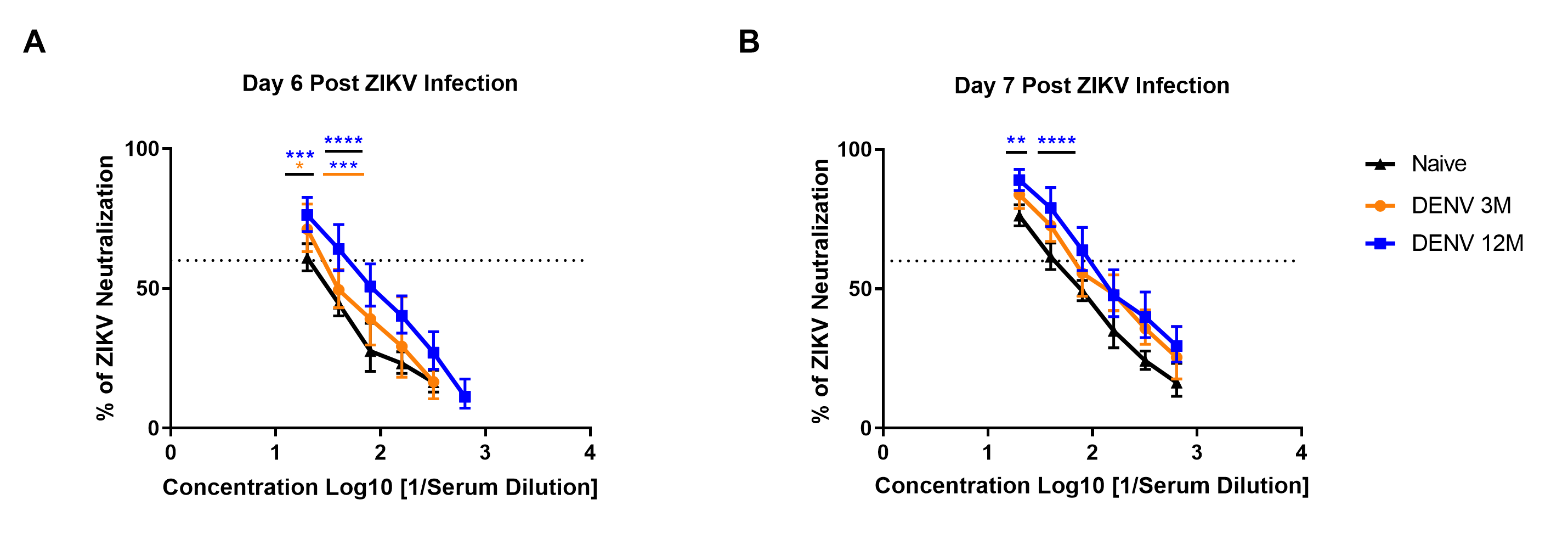

Supplement: S5 Fig — Dilution titers against ZIKV during days 6 and 7 post ZIKV infection. Animals from cohort 1 are shown in blue, animals from cohort 2 are shown in orange and naïve animals from cohort 3 are shown in black in all panels. Dotted line indicates the limit of detection for the assay. Non-neutralizing sera were assigned a value of one-half of the limit of detection for visualization and calculation of the geometric means and confidence intervals. Statistically significant differences among groups were calculated by two-way ANOVA using Tukey’s multiple comparisons test (*P<0.05, **P<0.001, ***P≤0.001 and ****P<0.0001). Colored stars represent a significantly different group, while colored lines represent the group that it is compared to. (TIF) [file pntd.0008285.s005.tif]

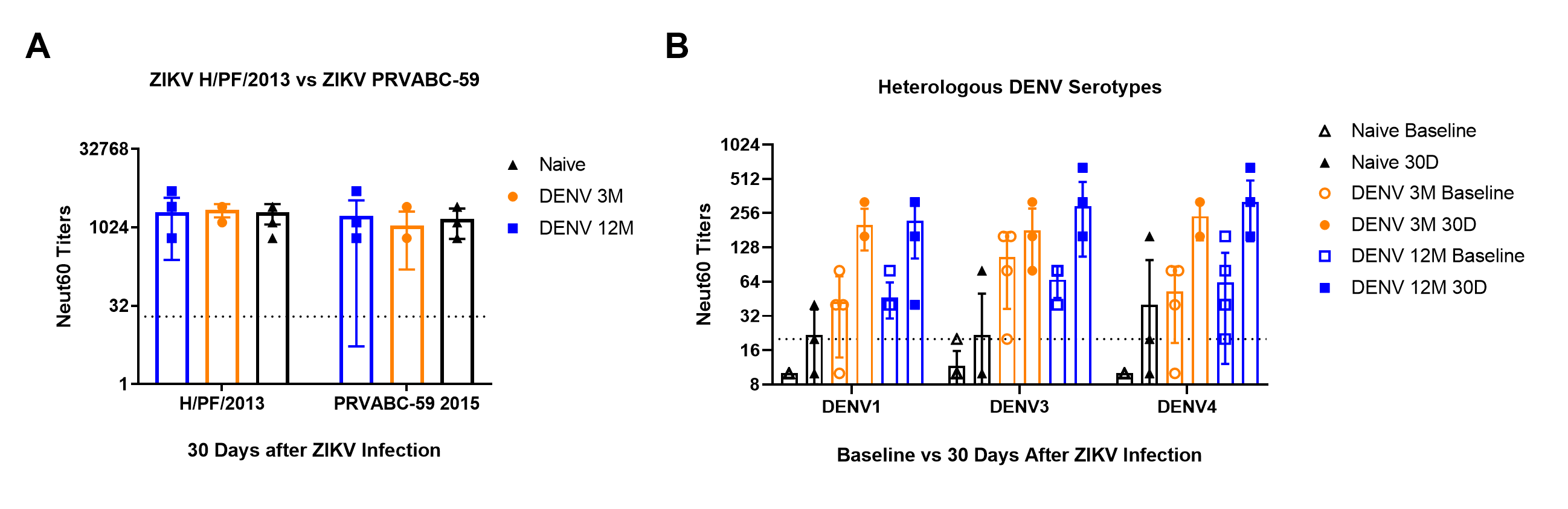

Supplement: S6 Fig — PRNT and FRNT assays were performed to determine the effect of previous DENV immunity in a subsequent ZIKV infection, and the neutralizing antibody response against different dengue serotypes and zika strains. In all panels, animals exposed to DENV 12 months before ZIKV infection are in blue, while animals exposed to DENV 3 months before are in orange. Naïve animals are in black. (A) Neutralization against two different ZIKV strains was performed. Dotted lines indicate the limit of detection for the assay. (B) Neutralizing response against heterologous DENV serotypes before and after ZIKV infection. (TIF) [file pntd.0008285.s006.tif]

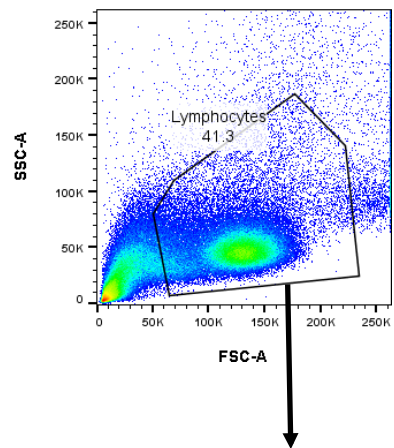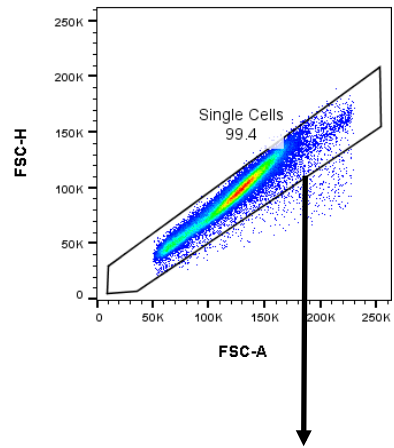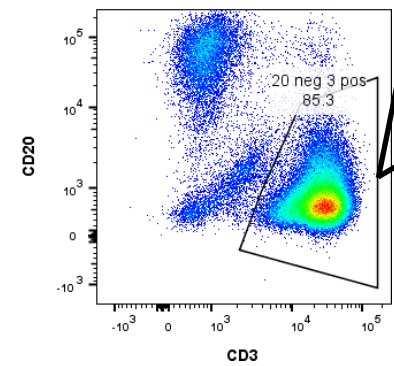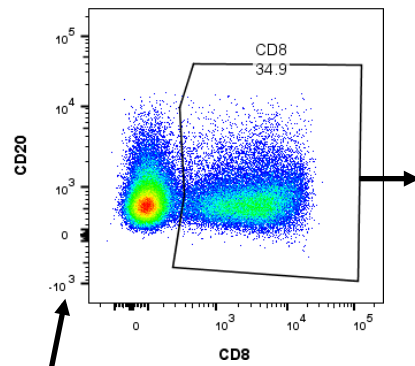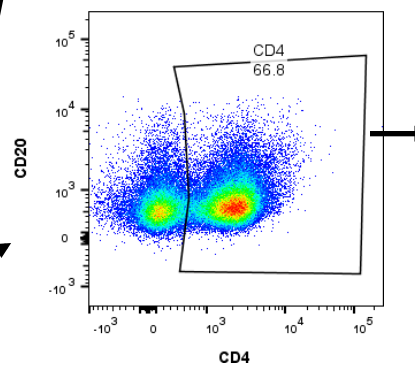

**Stimulated**

**Unstimulated**

**Stimulated**

**Unstimulated**

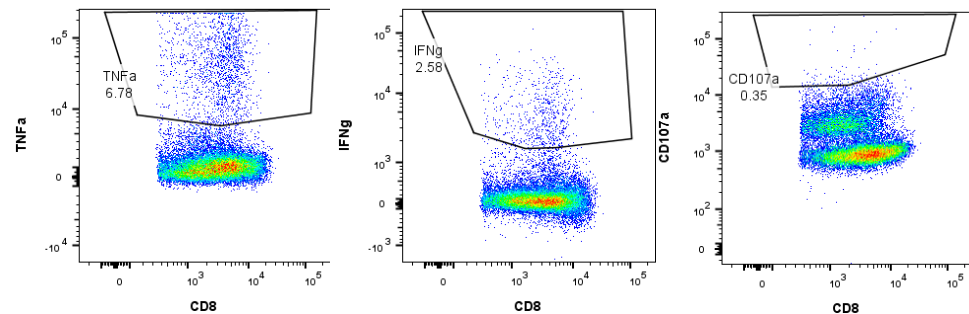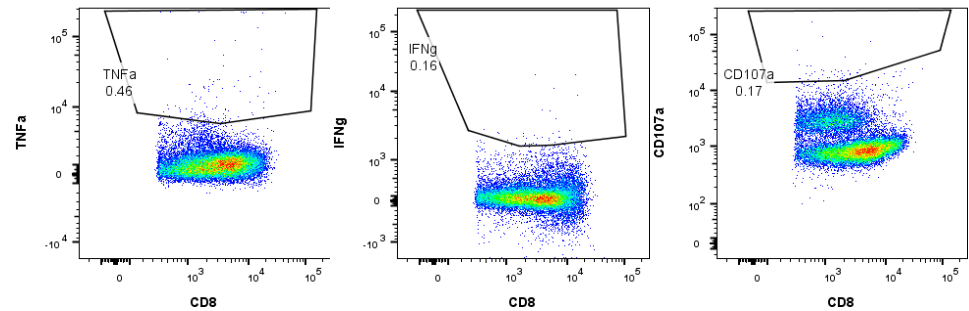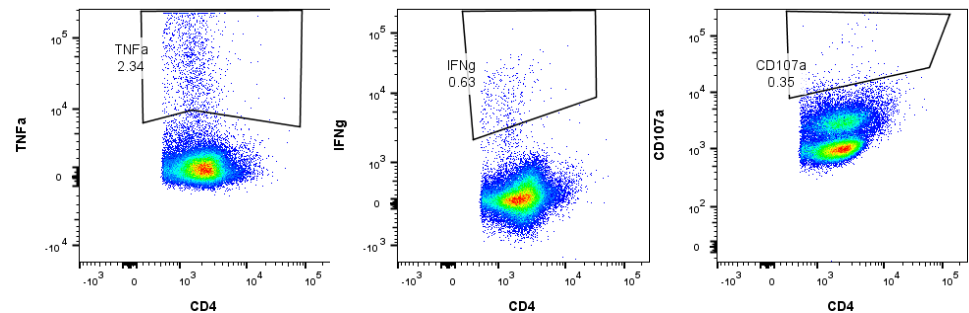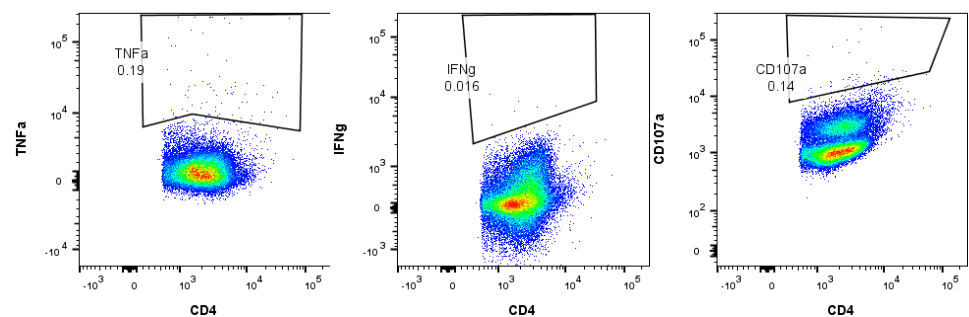

Supplement: S7 Fig — The gating strategy used to select cells expressing TNFa, IFNg and CD107a upon stimulation with various DENV and ZIKV peptides is shown. (PDF) [file pntd.0008285.s007.pdf]

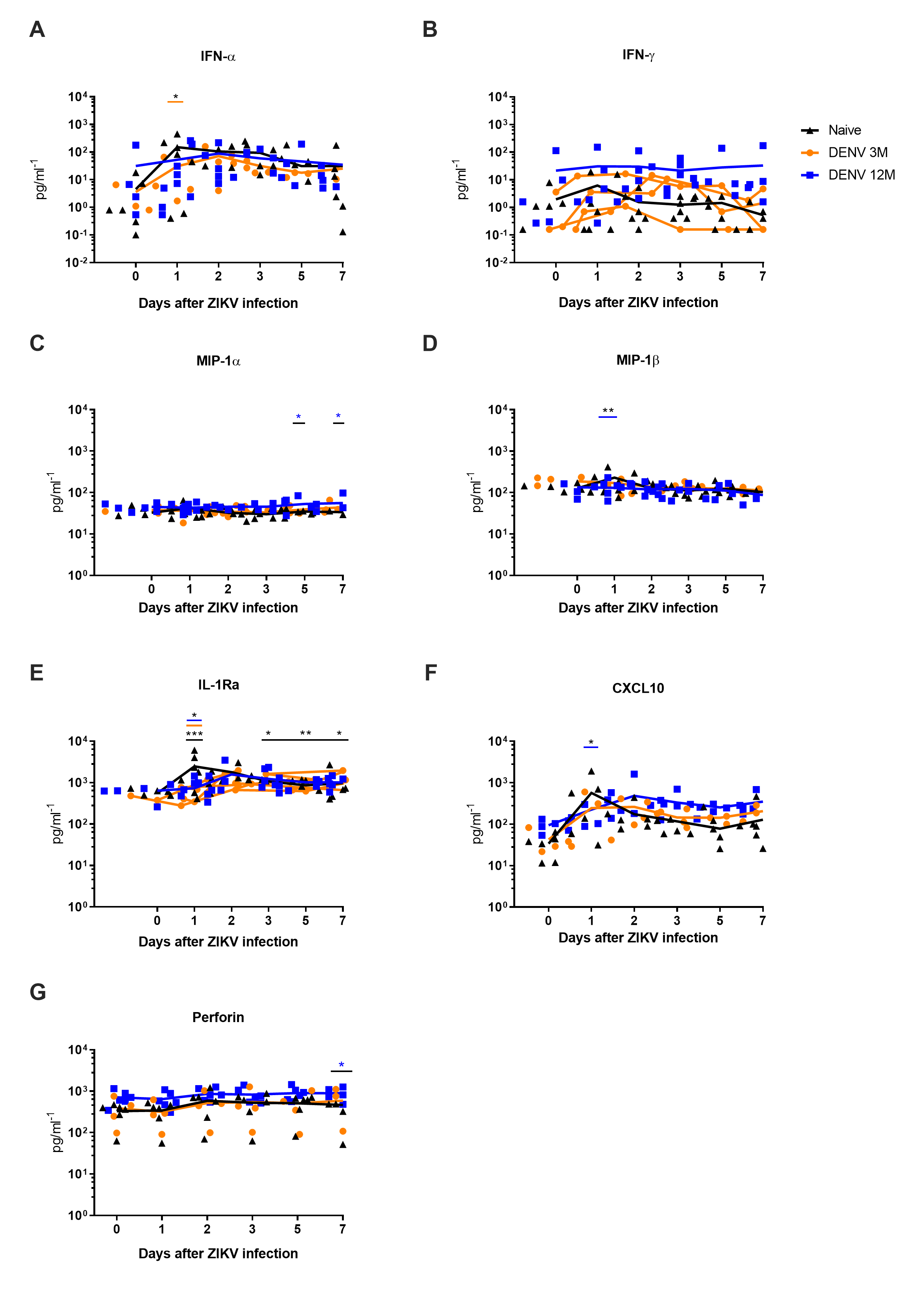

Supplement: S8 Fig — (A-G) Significant cytokine and chemokine profiles of are depicted in pg per ml-1. In all panels, animals exposed to DENV 12 months before ZIKV infection are in blue, while animals exposed to DENV 3 months before are in orange. Naïve animals are in black. Statistically significant differences among groups were calculated by two-way ANOVA using Tukey’s, Sidak’s and Dunnett’s multiple comparisons tests (*P<0.05, **P<0.001 and ***P≤0.0001). Colored stars represent a significantly different group, while colored lines represent the group that it is compared to. Same colored lines and stars represent a significant difference compared to their baseline. (TIF) [file pntd.0008285.s008.tif]

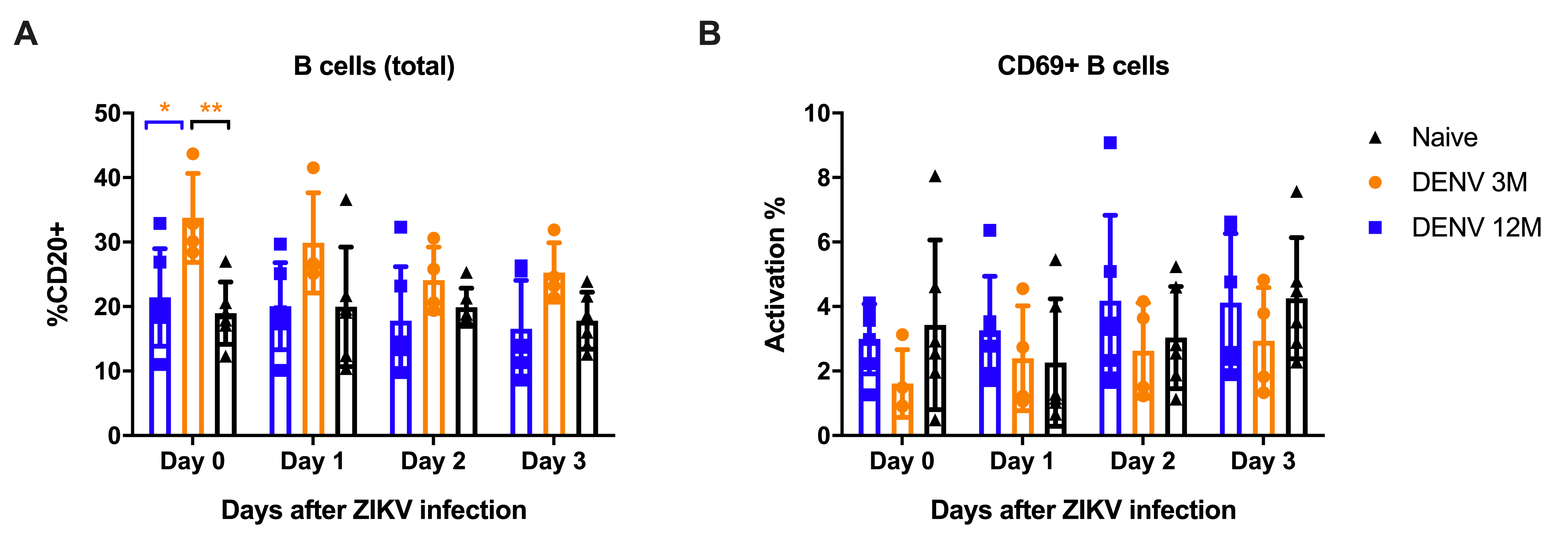

Supplement: S9 Fig — Frequency of B cells was assessed. In all panels, animals exposed to DENV 12 months before ZIKV infection are in blue, while animals exposed to DENV 3 months before are in orange. Naïve animals are in black. (A) Percentage of total B cells (CD20+) during baseline and days 1 through 3 p.i. (B) Frequency of activated B cells (CD20+ CD69+) during baseline and days 1 through 3 p.i. Comparisons between cohorts were performed by two-way ANOVA using Tukey’s multiple comparisons test (*P<0.05 and **P<0.01). Colored stars represent a significantly different group, while colored lines represent the group that it is compared to. (TIFF) [file pntd.0008285.s009.tiff]

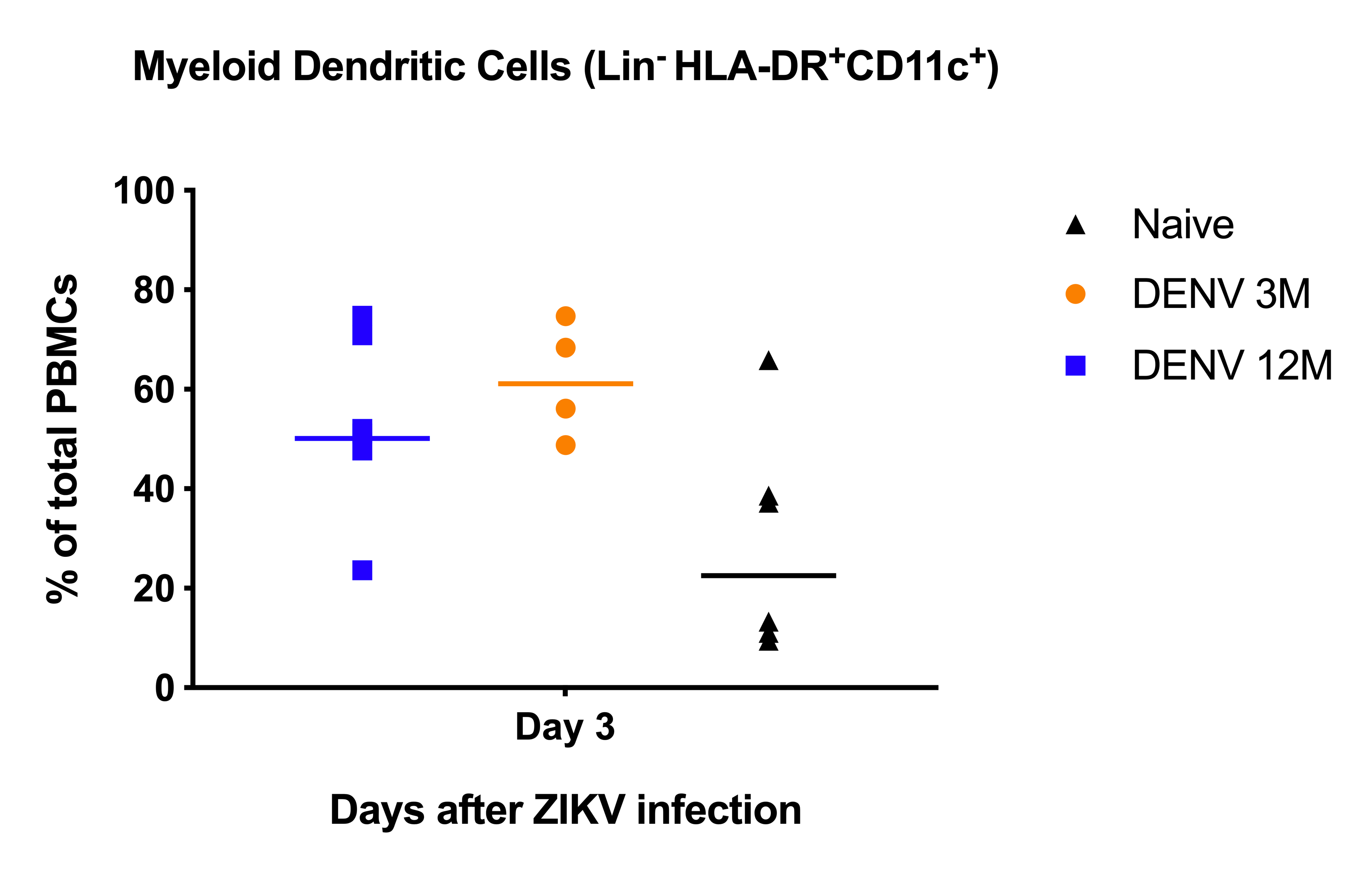

Supplement: S10 Fig — Percentage of myeloid lineage dendritic cells out of total gated PBMCs. Animals exposed to DENV 12 months before ZIKV infection are colored blue, while animals exposed 3 months before are colored orange. Naïve animals are depicted in black. No statistical differences were detected. (TIFF) [file pntd.0008285.s010.tiff]

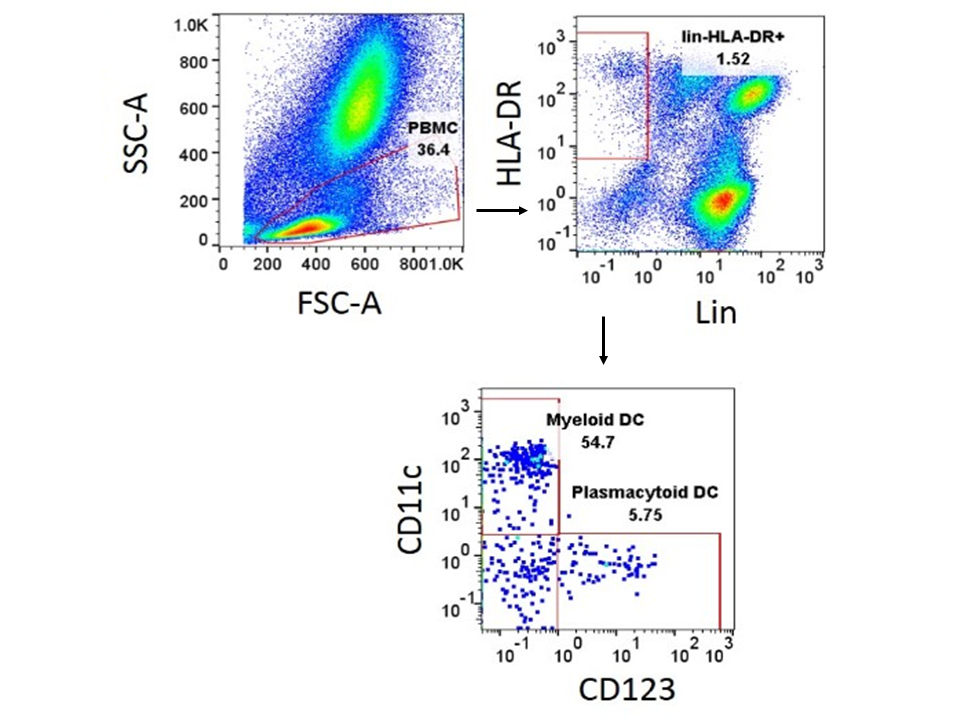

Supplement: S11 Fig — Gating strategy used to define dendritic cell subsets. (TIF) [file pntd.0008285.s011.tif]
